# Supplementary material for: Genetic and lifestyle modifiers of haemochromatosis-related clinical outcomes in HFE C282Y homozygotes
Source: JHEP Rep. 2026 Feb 13;8(4):101781. doi: 10.1016/j.jhepr.2026.101781 (PMC13019597; doi:10.1016/j.jhepr.2026.101781)
Supplement: Multimedia component 1 [file mmc1.pdf]

Genetic and lifestyle modifiers of haemochromatosis-related clinical outcomes in *HFE* C282Y homozygotes

Mitchell R Lucas, João Delgado, Robin N Beaumont, Gareth Hawkes, Andrew R Wood, Caroline F. Wright, Jeremy D Shearman, Janice L Atkins, Luke C Pilling

Table of contents

Supplementary methods 2

Supplementary tables.. separate excel file

Fig. S1 4

Fig. S2 5

Fig. S3 6

Fig. S4 7

Fig. S5 8

Supplementary methods

Polygenic Score for Iron Biomarkers

Supplementary Table 2 includes the genetic variants used to create the polygenic scores for each trait. The position is in human genome build 38. We used R package {ukbrapR} v0.3.5 to create the polygenic scores in the UK Biobank Research Analysis Platform (https://github.com/lcpilling/ukbrapR). This uses `bgenix` (https://enkre.net/cgi-bin/code/bgen/doc/trunk/doc/wiki/bgenix.md) to extract the variant calls from the UK Biobank whole genome sequencing DRAGEN variant calls (BGEN format, field:24309). Then `plink2` (https://www.cog-genomics.org/plink/2.0/) converts the subsetted BGEN file to BED format. Then `plink` v1.9 allele scoring function (https://www.cog-genomics.org/plink/1.9/score) is used to create the weighted allele score.

Baseline assessment (2006-2010)

We calculated body mass index (BMI) as weight (kg, field: 21002) divided by height (m^2, field: 50), waist-hip-ratio (WHR) by dividing waist circumference (cm, field: 48) by hip circumference (cm, field: 49). WHR was categorized into a binary variable based on sex-specific thresholds for central obesity.(24) Participants with WHR ≥0.96 (males) or WHR ≥0.85 (females) were assigned a value of 1, indicating high WHR, while those below these thresholds were assigned 0. Alcohol intake (fields: 1568 [weekly red wine], 1578 [weekly white wine/champagne], 1588 [weekly beer/cider], 1598 [weekly spirits], 1608 [weekly fortified wine], 4407 [monthly red wine], 4418 [monthly white wine/champagne], 4429 [monthly beer/cider], 4440 [monthly spirits], 4451 [monthly fortified wine], 4462 [monthly other alcoholic drinks], 5364 [weekly other alcoholic drinks]) was categorised by the number of units drank per week groups into '0 units per week', 1-14 units per week' (reference group, based on UK national recommendations1), '15-29 units per week' and 'over 30 units per week'. Smoking status (field: 20116) was defined as 'current smoker' based on subjects being asked "Do you smoke tobacco now? And grouped in a binary variable of 0 = no and 1 = yes, on most or all days' and 'Only occasionally'. Each participant was asked separate questions about their meat consumption during the assessment. They were specifically asked how often they ate processed meat (field: 1349), lamb/mutton (field: 1379), pork (field: 1389), and beef (field: 1369). For each type of meat, participants could choose from the following options: 'Never,' 'Less than once a week,' 'Once a week,' '2-4 times a week,' '5-6 times a week,' or 'Once or more daily'. We provided a value for each respective response on meat consumption: (Never = 0) (Less than once a week = 0.5) (Once a week = 1) (2-4 times a week = 3) (>4 times a week = 5.5) then derived a summed total weekly consumption of red /processed meat and recorded

as follow ‘0 times/week’ (reference group) and ‘0.1-2.9 times/week’, and ‘≥3.0 times/week’.(25) Missing assessment data were not imputed: complete-case analyses were used throughout.

Fig. S1

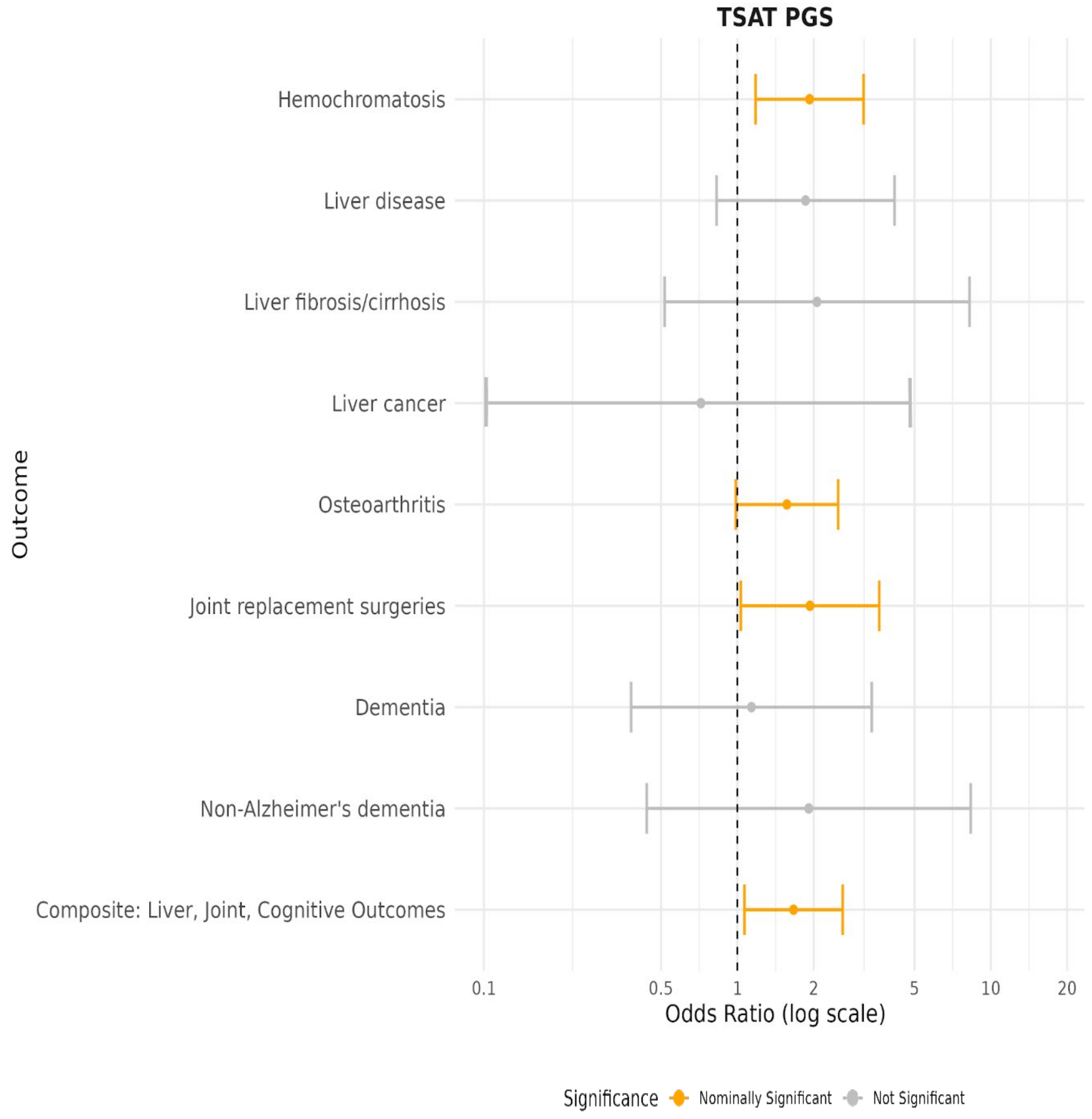

Associations between the highest quintile of TSAT polygenic biomarker and outcomes in C282Y homozygous male UK Biobank participants, without baseline hemochromatosis

Fig. S2

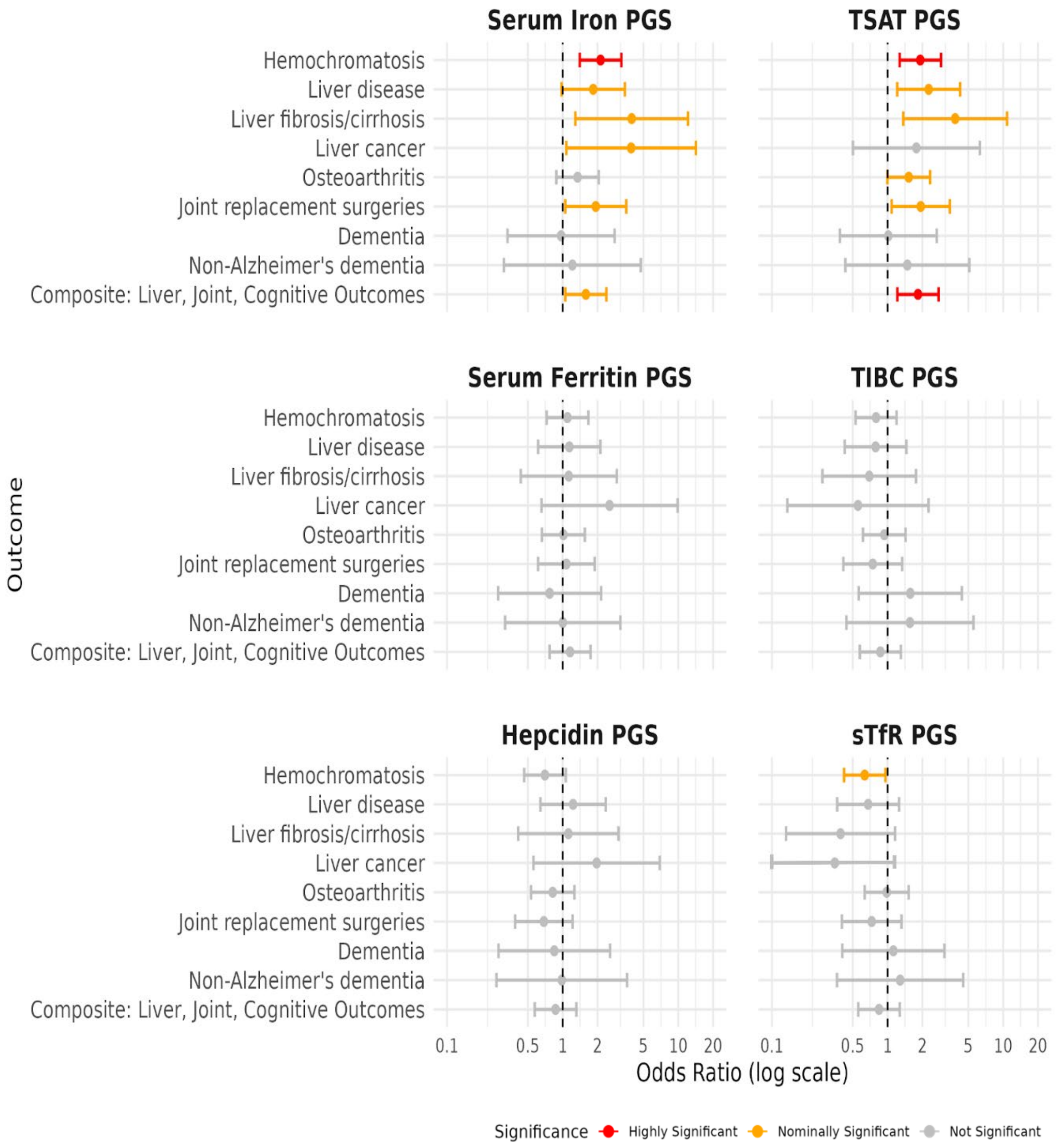

Associations between the highest quintile of iron polygenic biomarkers and outcomes in C282Y homozygous male UK Biobank participants.

Fig. S3

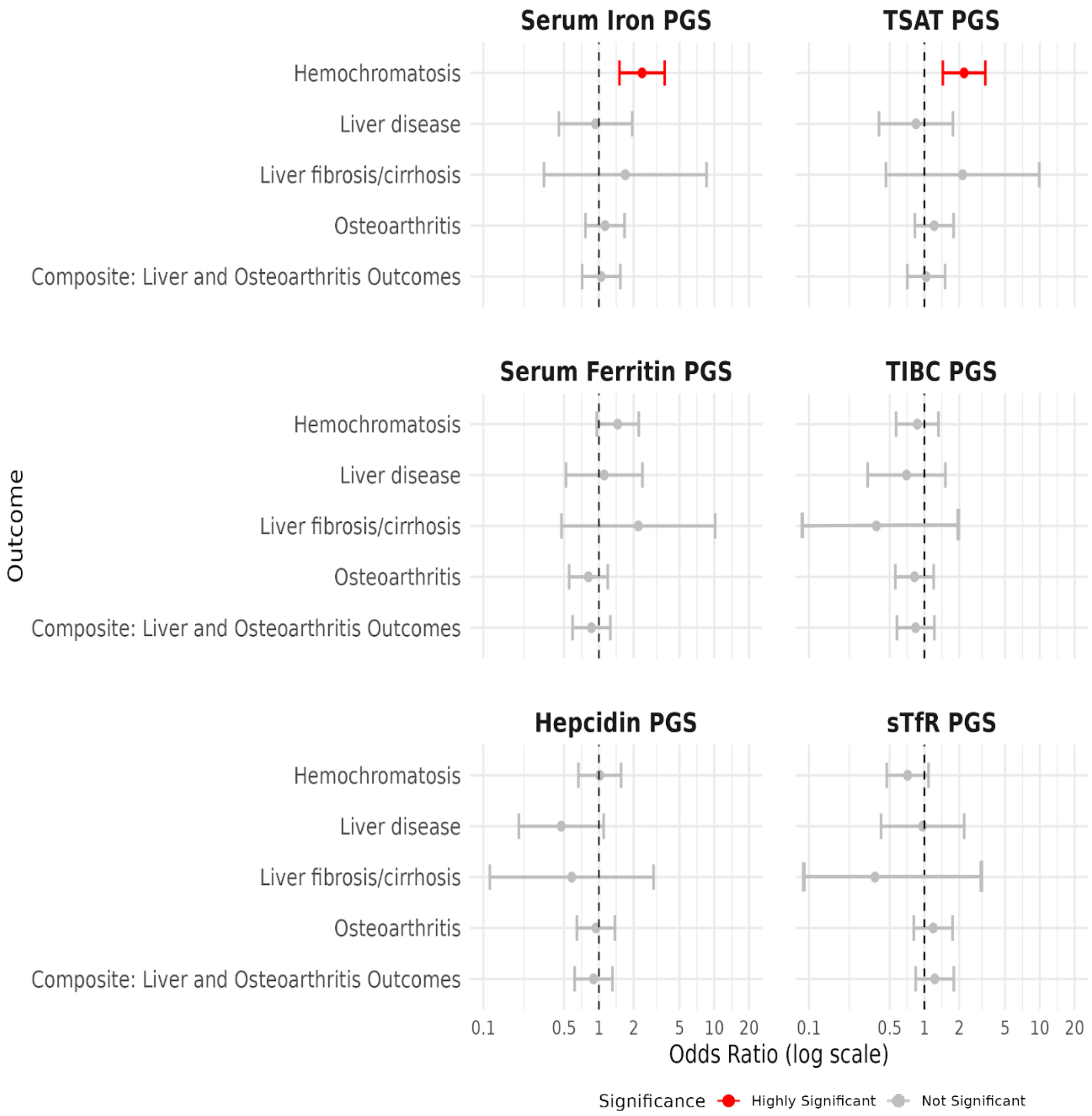

Associations between the highest quintile of iron polygenic biomarkers and outcomes in C282Y homozygous female UK Biobank participants

Fig. S4

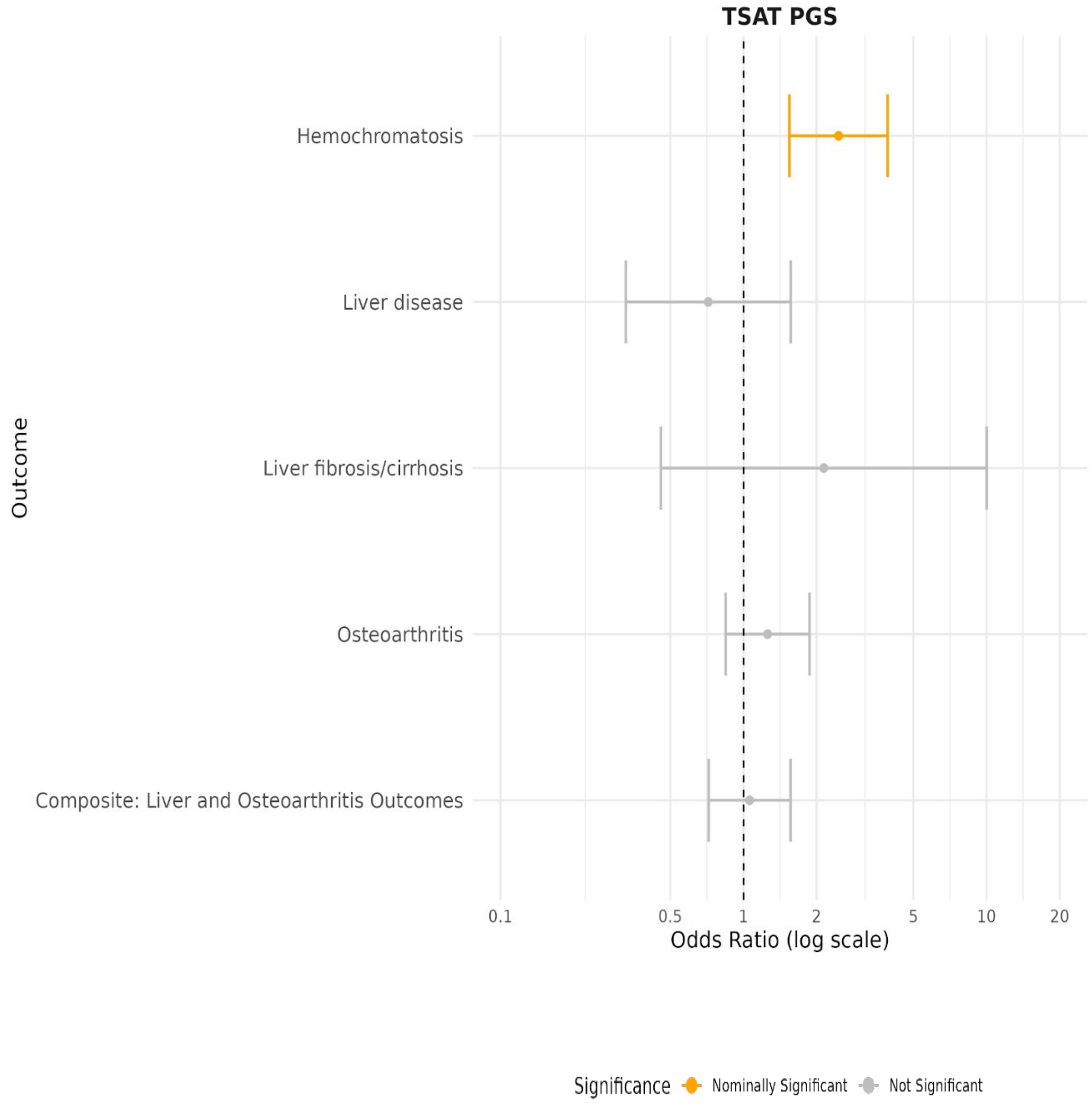

Associations between the highest quintile of TSAT polygenic biomarker and outcomes in C282Y homozygous female UK Biobank participants, without baseline hemochromatosis.

Fig. S5

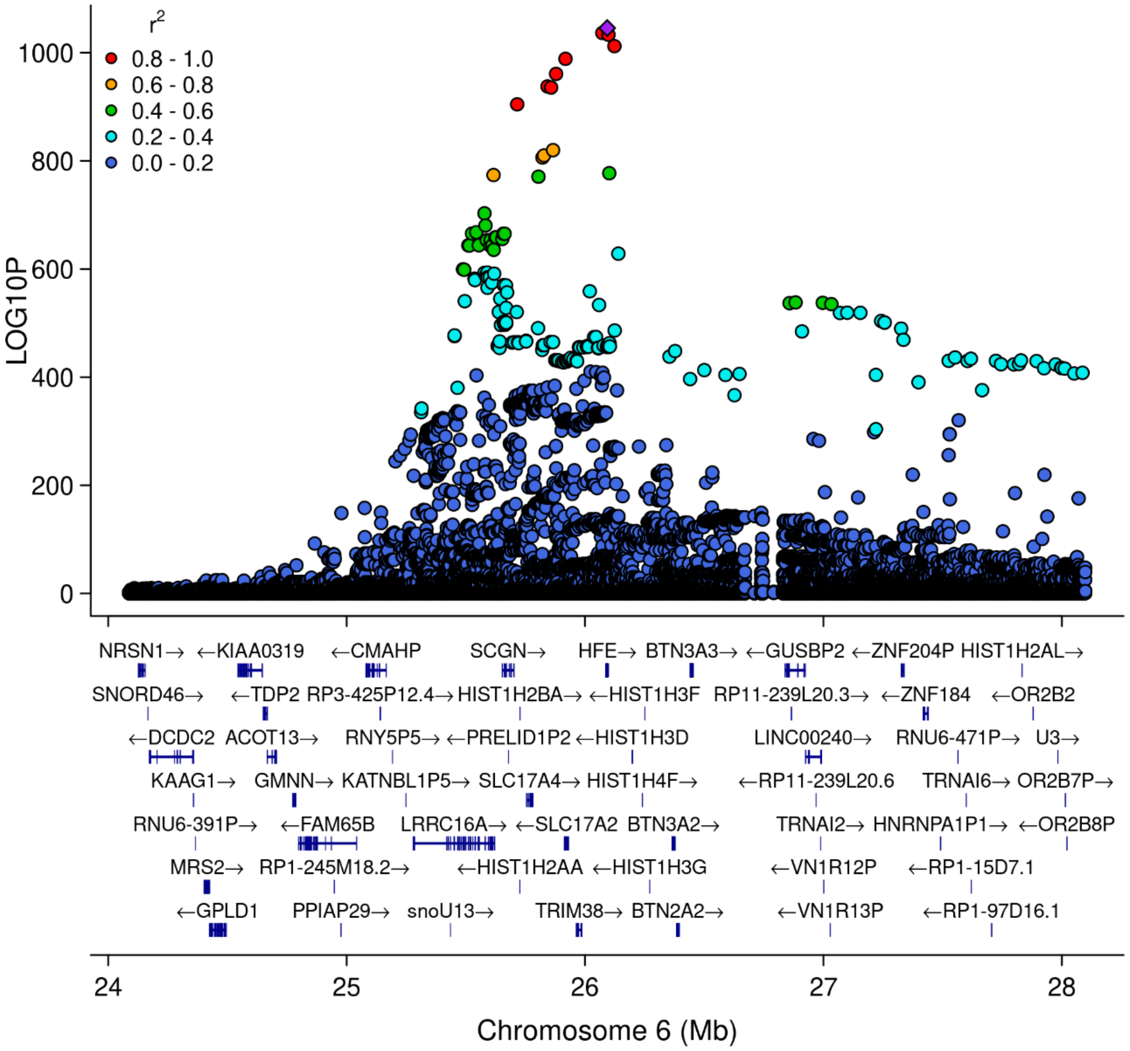

LocusZoom plot of *HFE* region on chromosome 6. Each point is a genetic variant: the x-axis shows it's location on chromosome 6, and the y-axis is the strength of association with haemochromatosis diagnosis (-$\log_{10}$ *p*-value). The points are coloured by their correlation coefficient (R^2) with *HFE* C282Y (rs1800562), coloured purple, in the UK Biobank EUR-like participants. Gene names and positions are indicated below the points, for reference. R package {locuszoomr} v0.3.8 was used to generate the plot (https://github.com/myles-lewis/locuszoomr).
