# Supplementary material for: Genetic and lifestyle modifiers of haemochromatosis-related clinical outcomes in HFE C282Y homozygotes
Source: JHEP Rep. 2026 Feb 13;8(4):101781. doi: 10.1016/j.jhepr.2026.101781 (PMC13019597; doi:10.1016/j.jhepr.2026.101781)
Supplement: Multimedia component 5 [file mmc5.pdf]

Genetic and lifestyle modifiers of haemochromatosis-related clinical outcomes in *HFE* C282Y homozygotes

Authors

Mitchell R. Lucas, João Delgado, Robin N. Beaumont, …, Jeremy D. Shearman, Janice L. Atkins, Luke C. Pilling

Correspondence

l.pilling@exeter.ac.uk (L.C. Pilling).

Graphical abstract

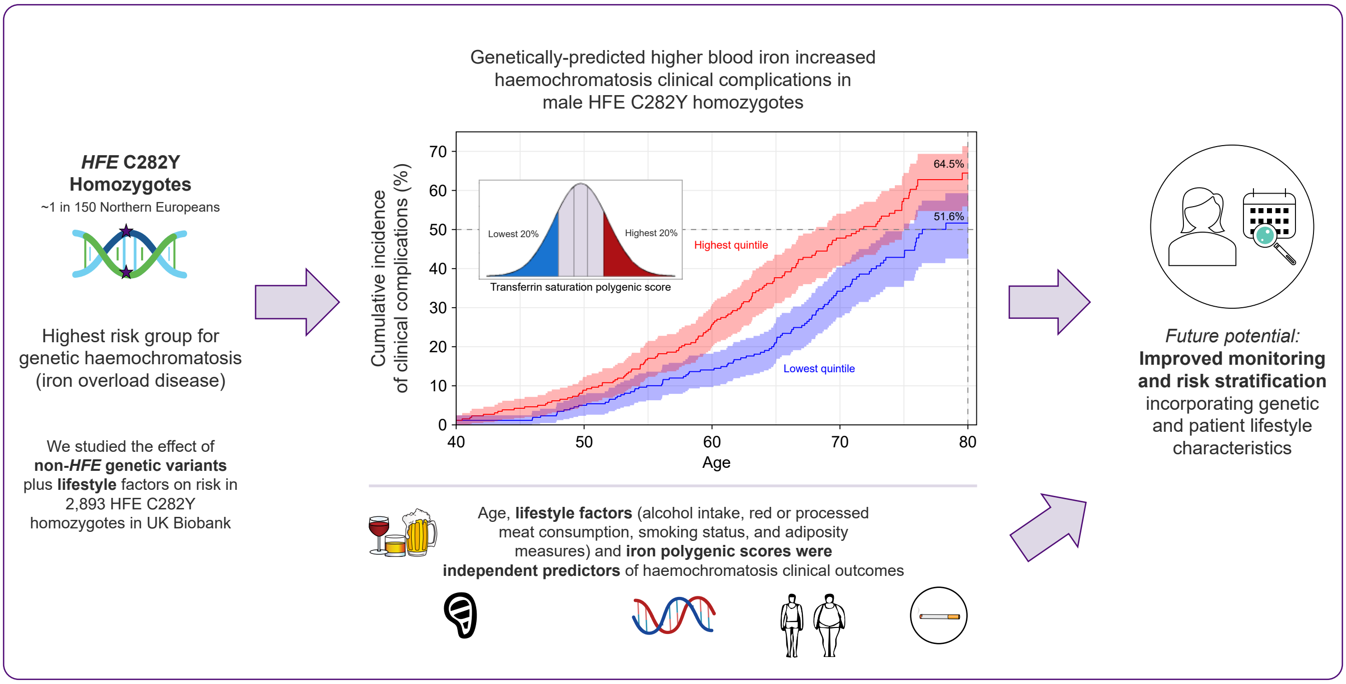

Highlights:

- There is a pressing clinical need to understand the wide variation in clinical outcomes observed in *HFE* C282Y homozygotes.
- Higher genetically predicted TSAT increased liver and musculoskeletal risks, highlighting non-*HFE* genetic influence on disease penetrance.
- Combining TSAT PGS with demographic and lifestyle factors gave the highest predictive accuracy.
- Polygenic risk may enable earlier targeted care, but external validation is required before clinical adoption.

Impact and implications:

There is a pressing clinical need to understand the wide variation in clinical outcomes observed in *HFE* C282Y homozygotes. Higher genetically predicted TSAT significantly increased the risk of clinical outcomes, including liver and musculoskeletal complications, in *HFE* C282Y homozygotes, highlighting non-*HFE* genetic influence on disease penetrance. These results are relevant for physicians and researchers, because combining genetic factors (TSAT PGS) with demographic and lifestyle factors provided the highest prediction accuracy for haemochromatosis and related clinical outcomes. Practically, integrating polygenic risk assessments with existing patient care pathways could enhance precision therapies by enabling the earlier, targeted management of high-risk *HFE* C282Y homozygotes, although external validation of these predictive models is required before clinical adoption.

https://doi.org/10.1016/j.jhepr.2026.101781
© 2026 The Author(s). Published by Elsevier B.V. on behalf of European Association for the Study of the Liver (EASL). This is an open access article under the CC BY license (http://creativecommons.org/licenses/by/4.0/).

Genetic and lifestyle modifiers of haemochromatosis-related clinical outcomes in *HFE* C282Y homozygotes

Mitchell R. Lucas[1], João Delgado[1], Robin N. Beaumont[1], Gareth Hawkes[1], Andrew R. Wood[1], Caroline F. Wright[1], Jeremy D. Shearman[2,3], Janice L. Atkins[1], Luke C. Pilling[1,*]

JHEP Reports **2026**. vol. 8 | 1–11

Check for updates

Background & Aims: The iron overload disease haemochromatosis is primarily caused by *HFE* p.C282Y homozygosity, yet penetrance of clinical outcomes (including liver disease/cancer) varies. We aimed to estimate the effect of genetic and lifestyle factors on disease penetrance and expressivity in *HFE* C282Y homozygotes.

Methods: We analysed 2,893 C282Y homozygous UK Biobank participants (n = 1,295 male). We ascertained haemochromatosis from medical records, liver disease/cancer, osteoarthritis, joint replacement surgeries, and dementia diagnoses. We derived polygenic scores (PGS) for iron biomarkers, including hepcidin and transferrin saturation (TSAT). Sex-stratified logistic regression assessed associations with clinical outcomes. We used time-to-event regression estimating effects of age, lifestyle, and PGS, and estimated effects of rare *HFE* variants using whole-genome sequencing data.

Results: In male *HFE* C282Y homozygotes, higher TSAT PGS increased the likelihood of diagnosis of haemochromatosis, and separately any clinical consequence (odds ratio $[\text{OR}]_{\text{top-}vs\text{-bottom-PGS-quintile}}$ = 1.83, 95% CI: 1.26–2.66, p = 0.001). Cumulative incidence of assessed haemochromatosis clinical outcomes in men by age 80 years was 64.5% (highest quintile) *vs.* 51.6% (lowest) (p for difference = 0.025). In women, TSAT PGS increased haemochromatosis likelihood (cumulative incidence: 45.3% *vs.* 23.3% [highest/lower quintile], p = 0.00001) but not liver disease. PGS for other iron biomarkers was not significantly associated with clinical outcomes. Rare heterozygous predicted loss-of-function variants in *HFE* increased haemochromatosis likelihood in non-C282Y homozygotes (aggregate OR = 14.8, 95% CI 4.7–41.1, p = 0.003), highlighting the importance of sequencing undiagnosed individuals to find rare causes of haemochromatosis.

Conclusion: Higher genetically predicted TSAT significantly increased risk of clinical outcomes in *HFE* C282Y homozygotes. Combined with modifiable lifestyle factors, genetic information could refine risk stratification and personalise iron monitoring, following validation.

© 2026 The Author(s). Published by Elsevier B.V. on behalf of European Association for the Study of the Liver (EASL). This is an open access article under the CC BY license (http://creativecommons.org/licenses/by/4.0/).

Introduction

HFE haemochromatosis is the most common genetic disorder among individuals of Northern European ancestry.[1] It is predominantly caused by *HFE* C282Y homozygosity, seen in one in 150 individuals of estimated European (EUR) genetic ancestry (C282Y allele frequency = 7%).[2] While the *HFE* H63D variant is more common (allele frequency 10–29%), its clinical manifestations are lower.[3] Haemochromatosis is characterised by iron overload, which significantly increases the risk of clinical outcomes,[4] including liver disease and hepatocellular carcinoma (HCC),[5] musculoskeletal disorders,[6] neurodegenerative diseases,[7] and all-cause mortality.[8] However, clinical penetrance among C282Y homozygotes varies.[9,10] In UK Biobank participants of EUR-like genetic ancestry, we estimated that 56% of C282Y homozygous men and 41% of C282Y homozygous women are expected to be diagnosed with haemochromatosis by age 80 years.[8] Although this is higher than many previous estimates, it suggests that other factors (genetic or lifestyle) modify phenotypic expression within C282Y homozygotes.[11,12]

HFE C282Y results in reduced hepcidin and increased iron absorption. Other iron metabolism genes include *HAMP, BMP2, FTL, SLC40A1, TMPRSS6*, and *TF*. Genetic variants in these genes are known to increase serum iron status biomarkers, such as serum ferritin (SF), transferrin saturation (TSAT), and iron.[13–18] Notably, *TF* polymorphisms explain ~40% of transferrin variation and mutations in *HJV* impair BMP signalling,[18] reducing hepcidin levels, and can also cause severe iron overload.[19] We previously reported an increased risk of liver disease in UK Biobank participants with higher genetically predicted TSAT.[11] In addition, we demonstrated that lifestyle factors and patient characteristics, such as alcohol intake, smoking, frequent consumption of red or processed meat, and obesity, were associated with higher levels of MRI-estimated liver iron in ~40,000 UK Biobank participants.[12]

* Corresponding author. Address: AGE group, University of Exeter, College House, Magdalen Road, Exeter, EX1 2LU, UK.
E-mail address: l.pilling@exeter.ac.uk (L.C. Pilling).
https://doi.org/10.1016/j.jhepr.2026.101781

In this study, we created weighted allele scores (polygenic scores [PGS]) for each participant to estimate their genetically predicted iron metabolism biomarkers, including novel predictors hepcidin, serum transferrin receptor (sTfR), as well as serum iron, TSAT, SF, and total iron-binding capacity (TIBC). This study substantially extends previous work by incorporating a longer follow-up and expanding the risk factors, genetics, and clinical outcomes examined. Using data from the UK Biobank, we aimed to clarify genetic and lifestyle modifiers of clinical penetrance in *HFE* C282Y homozygotes. By using PGS related to iron metabolism biomarkers and key lifestyle characteristics, we quantified their combined predictive value for clinically meaningful outcomes. We also investigated the effect of rare genetic mutations in *HFE* in non-C282Y homozygotes, aiming to determine novel genetic risk factors for haemochromatosis.

Patients and methods

Study population

UK Biobank includes 502,464 community volunteers with baseline ages 39–73 years, recruited from 22 centres in the UK (England, Scotland, and Wales) from 2006 to 2010. Participants are moderately healthier than the general population;[20] however, *HFE* allele frequencies are comparable to other UK-based studies.[5] Our studied sample included participants (n = 450,401) genetically similar to the 1000 Genomes Project European Ancestry superpopulation ('EUR-like'),[21] the definition of this population has been described previously.[22] *HFE* C282Y (rs1800562 G>A; NM_000410.3[HFE]:c.845G>A [p.Cys282Tyr]) and H63D (rs1799945 C>G; NM_000410.4[HFE]:c.187C>G [p.His63Asp]) genotype information was from the whole-genome sequencing DRAGEN variant calls (UK Biobank field: 24310[23]). UK Biobank obtained ethics approval from the Northwest Multicenter Research Ethics Committee (reference: 11/NW/0382). Informed consent was obtained from all participants. Consent did not include feedback to participants on genotype. Access was granted under UK Biobank approved applications 14631 and 103356.

Baseline assessment (2006–2010)

For detailed derivations, please see the supplementary data. In brief: we calculated BMI as weight (kg) divided by height (m^2) and waist–hip ratio (WHR) as waist circumference (cm) divided by hip circumference (cm). WHR was categorised into a binary variable based on sex-specific thresholds (WHR ≥0.96 [men] or WHR ≥0.85 [women]) for central obesity.[24] Alcohol intake was the number of units consumed per week ('0 units per week', '1–14 units per week' [reference group], '15–29 units per week' and '>30 units per week'). Smoking status was defined as current, former, or never smoked. Each participant was asked about their meat consumption, specifically how often they ate processed meat, lamb/mutton, pork, and beef. We classified the participants as follow: '0 times/week', '0.1–2.9 times/week', and '≥3.0 times/week'.[25] Missing assessment data were not imputed: complete-case analyses were used throughout.

Diagnosis ascertainment

England, Wales, and Scotland hospital inpatient admission records (Hospital Episode Statistics [HES]) were available from April 1996 to October 2022. Incident diagnoses and surgical procedures were from hospital inpatient data (baseline to October 2022) plus cancer registries to December 2020 for England and Wales and November 2021 for Scotland. Corresponding primary care diagnosis codes were identified using the UKB 'Clinical Coding Classification Systems and Maps' resource to map ICD-10 codes to Read2/CTV3 (UK Biobank resource: 592). Outcomes ascertained were haemochromatosis, liver disease, liver fibrosis or cirrhosis, liver cancer, osteoarthritis, joint replacement surgeries, dementia, and Alzheimer's disease, non-Alzheimer's dementia (see Table S1 for ascertainment codes for all outcome diagnoses). Prevalent diagnoses also included data from baseline self-report information (fields: 20001 and 20002). We used R package {ukbrapR} v0.3.5 to ascertain diagnoses in the UK Biobank Research Analysis Platform (https://github.com/lcpilling/ukbrapR). 'Joint replacement surgeries' included hip, knee, ankle, or shoulder replacement surgery.

Polygenic score for iron biomarkers

We used data from the UK Biobank whole-genome sequencing DRAGEN variant calls (BGEN format, field: 24309)[23] to create PGS for six iron-status biomarkers (hepcidin, sTfR, serum iron, TSAT, SF, and TIBC), among 451,046 participants of EUR genetic ancestry. For hepcidin and sTfR, we used 43 independent genetic variants identified in a genome-wide association meta-analysis of up to 91,675 participants for hepcidin and 45,330 participants for sTfR, from 12 European-based cohorts (CHRIS, CROATIA_Vis, Danish Blood Donor Study, deCODE, FinDonor 10,000 Studies 1 and 2, InCHIANTI, INGI-Val Borbera, INTERVAL, KORA_F3, Nijmegen Biomedical Study, and PREVEND). The analysis identified 15 novel loci associated with hepcidin and sTfR concentrations.[26] For serum iron, TSAT, SF, and TIBC, we used 128 independent genetic variants identified in a genome-wide association study (GWAS) of 257,953 individuals from six cohorts:[27] the Trøndelag Health Study (HUNT), Michigan Genomics Initiative (MGI), SardiNIA, deCODE (Iceland), Interval Study (UK), and the Danish Blood Donor Study (DBDS). The study population was predominantly of EUR genetic ancestry. These included 20 variants linked to iron levels, 64 to ferritin, 19 to TSAT, and 41 to TIBC. Given that genetic variants remain stable throughout life, PGS serves as a measure of lifelong genetic risk; individuals with higher PGS (*e.g.* genetically predicted higher serum iron levels) have higher lifetime susceptibility to higher levels[28] (see Table S2 for variants included). We used R package {ukbrapR} v0.3.5 to create the polygenic scores in the UK Biobank Research Analysis Platform (https://github.com/lcpilling/ukbrapR). In brief, this uses `bgenix` (https://enkre.net/cgi-bin/code/bgen/doc/trunk/doc/wiki/bgenix.md) to extract the variant calls from the UK Biobank whole-genome sequencing DRAGEN variant calls (BGEN format, field:24309) and `plink2` (www.cog-genomics.org/plink/2.0/) to convert the subsetted BGEN file to PGEN format and create the weighted allele score.

Analysis of rare variants in *HFE*

We used data from the UK Biobank whole-genome sequencing DRAGEN variant calls (BGEN format, field: 24309)[23] in the 451,046 participants of EUR genetic ancestry and REGENIE v3.3 to estimate associations between genetic

variants in the *HFE* gene region (±2 Mbp of *HFE*: human genome build GRCh38 coordinates chr6:24087525-28098445) and diagnosed haemochromatosis, adjusted for age, sex, relatedness, and population stratification. The analysis was performed: (1) at the single-variant level for variants with minor allele count ≥10; and (2) by aggregating rare variants (minor allele frequency <0.001) with similar predicted consequence (*e.g.* 'predicted loss-of-function' (pLoF', including frameshift, stop gain, and splice acceptor/donor variants) or 'missense' variants (aggregate variant analysis increases power compared with single variant analysis). Sensitivity analyses were conditioned on the *HFE* C282Y genotype to adjust for any linkage disequilibrium (*i.e.* to identify novel haemochromatosis causes beyond the known genotypes).

Statistical analysis

We first analysed each iron biomarker PGS as a continuous variable within *HFE* C282Y homozygous participants. Each PGS was standardised to z-scores (mean = 0, SD = 1) before analyses to ensure interpretability of effect estimates across models and between PGS. Sex-stratified logistic regression models, adjusted for age, assessment centre, and genetic principal components (PC1–PC10), were used to evaluate associations between standardised PGS and clinical outcomes. Genetic PCs were included to control for population stratification bias.[29]

Next, *HFE* C282Y homozygous participants were categorised into quintiles based on each iron biomarker PGS. We conducted sex-stratified logistic regression analyses comparing clinical outcomes between the highest and lowest quintiles, adjusting again for age, assessment centre, and PC1–PC10.[29]

Clinical outcomes included in this analysis were selected based on our previously published study,[8] which identified specific outcomes that were significantly (false discovery rate [FDR]-adjusted *p* <0.05) increased in risk within male or female C282Y homozygotes compared with individuals without *HFE* C282Y or H63D variants. These were: haemochromatosis, liver disease, liver fibrosis/cirrhosis, liver cancer, osteoarthritis (both sexes), joint replacement surgeries, dementia, and non-Alzheimer's dementia in male C282Y homozygotes; and haemochromatosis, liver disease, liver fibrosis/cirrhosis, and osteoarthritis in women. We aggregated haemochromatosis-associated clinical outcomes into a composite outcome termed 'Any outcome' throughout (to clarify, haemochromatosis cases were not excluded from the primary analysis, but the 'Any outcome' included haemochromatosis-associated clinical complications).

In this analysis, we applied Benjamini–Hochberg (BH) FDR correction. The BH-FDR correction was applied separately to tests conducted in men (n = 4) and women (n = 30), because no direct comparisons between sexes were performed. *p*-values were ranked, and FDR-adjusted significance thresholds (q = 0.05) were determined to control the expected proportion of false discoveries.

Kaplan-Meier analyses estimated cumulative incident probabilities for outcomes, comparing the lowest and highest PGS quintiles from age 40 to 80 years, stratified by sex. To assess differences in cumulative incidence at specific time points (*e.g.* age 80 years), we compared survival probabilities between groups using a Z-test based on Greenwood's standard errors.

For predictive modeling, we used time-to-event (Cox regression) models from baseline assessment to the event date or date of censoring (30 October 2022). Participants with prevalent diagnoses of clinical outcomes (assessed previously in logistic regression models) at baseline were excluded to maintain temporality between baseline measurements (lifestyle factors and genetic predictors) and subsequent clinical outcomes, in accordance with Bradford Hill's temporality criterion for causality.[30] We compared the Harrell's C-statistic between models to evaluate relative predictive performance of age, iron-related lifestyle factors (alcohol intake, smoking, red/processed meat consumption, WHR, and BMI), and iron biomarker PGS individually and in combination, stratified by sex. No further validation (*e.g.* bootstrapping of C-statistics) was performed.

We also performed sex-stratified analysis of serum iron and TSAT polygenic scores on haemochromatosis separately in other *HFE* C282Y and H63D genotype groups (H63D heterozygotes, H63D homozygotes, C282Y–H63D compound heterozygotes, C282Y heterozygotes, and individuals without C282Y or H63D variants).

Unless otherwise specified, we used R version 4.1.1 and REGENIE version 3.3 on the UK Biobank Research Analysis Platform for analysis. Primary analyses were performed between November 2024 and June 2025.

Sensitivity analysis

In sensitivity analyses we excluded those diagnosed with haemochromatosis at baseline assessment to avoid bias from those likely undergoing iron monitoring and treatment or who might have received treatment shortly after baseline. We also assessed the potential impact of familial relatedness by removing participants related to the third degree or closer. Finally, associations with rare *HFE* pLoF variants were explored, highlighting their independent contribution to risk of haemochromatosis, liver disease, liver cancer, osteoarthritis, and dementia, adjusting for age, sex, PC1–PC5, and known *HFE* haemochromatosis-associated genotypes (C282Y and H63D).

Results

Participant characteristics

We studied 1,295 male and 1,598 female *HFE* C282Y homozygotes. The mean age at baseline assessment was 56.9 years (SD 8.1). 36.1% of men and 26% of women had been diagnosed with haemochromatosis by the date of censoring (31 October 2022). We studied clinical outcomes identified previously to be associated with C282Y homozygosity, including any liver disease, liver fibrosis or cirrhosis, liver cancer, 'any liver disease', osteoarthritis, dementia, and joint replacement surgeries. We found that 41.8% of men and 35.9% of women had any of the associated clinical complications (Table 1). We also found sex differences in participant characteristics, including adiposity (BMI and WHR), alcohol and smoking behaviours, and red/processed meat consumptions (Table 1).

We assessed multicollinearity among the iron PGS biomarkers using correlation matrices and variance inflation factors (VIFs). Serum iron and TSAT PGSs were highly correlated

Table 1. Baseline characteristics of the UK Biobank *HFE* C282Y homozygote participants of European ancestry, stratified by sex.

Characteristics	All C282Y homozygotes (N = 2,893)	Male C282Y homozygotes (n = 1,295)	Female C282Y homozygotes (n = 1,598)
Age at baseline, mean (SD), years	56.9 (8.1)	57.0 (8.0)	56.8 (8.2)
Haemochromatosis, n (%)	836 (28.9)	468 (36.1)	368 (23.0)
Liver disease, n (%)	246 (8.5)	153 (11.8)	93 (5.8)
Liver fibrosis/cirrhosis, n (%)	86 (3.0)	64 (4.9)	22 (1.4)
Liver cancer, n (%)	N/A	35 (2.7)	N/A
Any replacement surgery, n (%)	N/A	169 (13.1)	N/A
Osteoarthritis, n (%)	937 (32.4)	411 (31.7)	526 (32.9)
Dementia, n (%)	N/A	51 (3.9)	N/A
Non-Alzheimer's dementia, n (%)	N/A	36 (2.8)	N/A
Any outcome*, n (%)	N/A	541 (41.8)	574 (35.9)
Alcohol intake, units/week, n (%)			
0	254 (9.0)	93 (10.1)	161 (7.2)
1–14	1,084 (37.5)	321 (47.7)	763 (24.8)
15–29	637 (22.0)	381 (29.4)	256 (16.2)
≥30	443 (15.2)	354 (27.3)	89 (5.6)
Missing	475 (16.4)	329 (20.6)	146 (11.3)
Red/processed meat consumption, times/week, n (%)			
0	146 (5.0)	41 (3.2)	105 (6.6)
0.1–2.9	1,050 (36.3)	354 (27.3)	696 (43.6)
≥3.0	1,697 (58.7)	900 (69.5)	797 (49.9)
Missing	0	0	0
Smoking status, n (%)			
Current	325 (11.2)	189 (14.6)	136 (8.5)
Previous	1,407 (36.2)	505 (39.0)	542 (33.9)
Never	1,512 (52.3)	595 (46.0)	917 (57.4)
Missing	9 (0.3)	6 (0.5)	<5
Waist-to-hip ratio, n (%)			
Normal	1,967 (68.1)	850 (65.6)	1,117 (69.9)
High	923 (31.9)	444 (34.3)	479 (30.1)
Missing	<5	<5	<5
BMI classification, kg/m^2, n (%)			
Underweight	18 (0.6)	5 (0.4)	13 (0.8)
Normal	979 (33.8)	333 (25.7)	646 (40.4)
Overweight	1,244 (43.0)	659 (50.9)	585 (36.6)
Obese	644 (22.2)	295 (22.8)	349 (21.8)
Missing	8 (0.3)	<5	5 (0.3)

Baseline characteristics and clinical outcomes among 2,893 *HFE* C282Y homozygotes. Outcomes were selected based on previous UK Biobank analyses showing statistically significant associations with C282Y homozygosity compared with individuals without C282Y or H63D variants (Bonferroni-adjusted *p* <0.0001), with a broader range observed in men.[8].

*Any outcome (not including haemochromatosis, although cases were not excluded) includes liver disease, liver fibrosis/cirrhosis, liver cancer, osteoarthritis, dementia, and joint replacement surgeries in men, and liver disease, liver fibrosis/cirrhosis, and osteoarthritis in women. N/A, outcomes not analysed because of lack of previous significant association.[8]

(r = 0.84), with VIFs of 7.66 and 12.66, respectively (Fig. 1). To address this, we excluded serum iron from subsequent multivariable models, based on evidence of high collinearity with TSAT, as indicated by both the correlation matrix and VIF diagnostics (Fig. 1). These analyses were conducted in the full UK Biobank EUR-like sample (n = 451,046).

After applying the BH FDR correction to account for multiple testing, significance thresholds for analyses using PGS as a continuous variable for male and female C282Y homozygotes became $p \leq 0.009$ and $p \leq 4 \times 10^{-6}$, based on 54 and 30 independent tests, respectively. Tables S3 and S4 detail the BH-FDR calculations. Analyses using PGS as highest *vs.* lowest quintiles were $p \leq 0.0014$ for men and $p \leq 6 \times 10^{-5}$ for women (Tables S5 and S6).

PGS and disease outcomes in male *HFE* C282Y homozygotes

Men with higher TSAT PGS showed significantly increased odds for haemochromatosis diagnosis (OR per 1-SD increase = 1.22, 95% CI: 1.09-1.38, *p* = 0.0009), liver disease (odds ratio [OR] = 1.30, 95% CI: 1.09–1.55, *p* = 0.004), liver fibrosis or cirrhosis (OR = 1.66, 95% CI: 1.27–2.20, *p* = 0.0003), and joint replacement surgeries (OR = 1.27, 95% CI: 1.07–1.51, *p* = 0.006) compared with those without a diagnosis of these clinical outcomes. Male C282Y homozygotes had increased odds of any haemochromatosis-associated morbidity (liver disease, liver cancer, osteoarthritis, joint replacement surgeries, or dementia)[8] (OR = 1.20, 95% CI: 1.07–1.35, *p* = 0.003) (Table S7).

Similarly, higher serum iron PGS was strongly associated with increased odds of haemochromatosis (OR per 1-SD increase = 1.30, 95% CI: 1.16–1.47, *p* = 0.00002), liver fibrosis or cirrhosis (OR = 1.57, 95% CI: 1.19–2.08, *p* = 0.002), liver cancer (OR = 1.75, 95% CI: 1.21–2.57, *p* = 0.004), and joint replacement surgeries (OR = 1.32, 95% CI: 1.11–1.57, *p* = 0.002). A significant association was also observed with any haemochromatosis-related outcome (OR = 1.22, 95% CI: 1.09–1.38, *p* = 0.0009). Serum iron PGS was not statistically associated with dementia outcomes (Table S7). Hepcidin, sTfR, SF, and TIBC PGS were not associated with outcomes after controlling for multiple tests ($p \leq 0.009$).

A

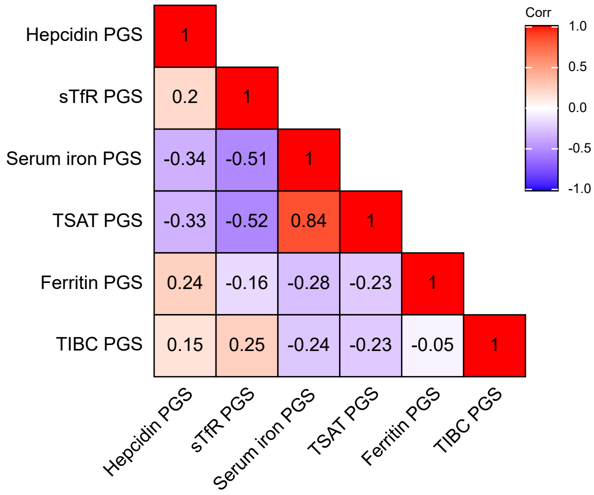

B

PGS predictor	VIF (before)	VIF (after)
Hepcidin	1.32	1.30
sTfR	1.37	1.35
Serum iron	7.66	-(removed)
TSAT	12.66	2.72
Ferritin (SF)	2.41	2.39
TIBC	1.55	1.51

Fig. 1. Pearson's correlation between iron status PGS. (A) Pearson correlations between PGS for six iron-related biomarkers within the whole UK Biobank EUR-like sample (n = 451,046). (B) VIF table illustrating multicollinearity diagnostics before and after the removal of serum iron. The decision to exclude serum iron was based on evidence of high collinearity with transferrin saturation, as indicated by both the correlation matrix and VIF values. EUR, European; PGS, polygenic score; VIF, variance inflation factor.

Men in the highest TSAT PGS quintile (top 20% for genetically predicted TSAT) had increased likelihood of haemochromatosis (OR = 1.92, 95% CI: 1.32–2.79, p = 0.0007) compared with those in the lowest quintile (Fig 2A), with a cumulative incidence by age 80 of 55.4% (95% CI: 46.4–62.8%) compared with 38.0% (29.4–45.5%]) with a statistical difference of p = 0.003 (see Table S8 for full list of male cumulative incidence results from age 40 to 80 years). TSAT PGS was also associated with increased likelihood of any haemochromatosis-associated morbidity (a composite outcome including liver disease, liver fibrosis/cirrhosis, liver cancer, osteoarthritis, dementia, non-Alzheimer's dementia, and joint replacement surgeries)[8] (OR = 1.83, 95% CI: 1.26–2.66, p = 0.001), with a cumulative incidence of 64.5% (95% CI: 55.9–71.3%) compared with 51.6% (42.6–59.3%) in the lowest quintile (p = 0.025) (Fig. 2A,B and Table S8). Effect sizes were slightly attenuated, excluding prevalent haemochromatosis diagnoses (Fig. S1 and Table S9). Nominal associations (p <0.05) were observed with diseases individually (liver disease, liver fibrosis/cirrhosis, osteoarthritis, and joint replacement surgeries; Fig. S2), although none were statistically significant after multiple testing correction (p >0.0014).

Similarly, men in the highest serum iron PGS quintile had significantly higher odds of being diagnosed with haemochromatosis (OR = 2.13, 95% CI: 1.46–3.10, p = 0.00008), with an estimated cumulative incidence to age 80 years of 59.3% (95% CI: 50.6–66.5%) compared with 37.6% (29.2–45.1%) in the lowest quintile (p = 0.00015) (see Table S10 for full list of ORs; Fig. 2 and Table S8). We found suggestive associations with liver disease (OR = 1.92, 95% CI: 1.07–3.46, p = 0.03), liver fibrosis/cirrhosis (OR = 3.96, 95% CI: 1.43–10.95, p = 0.008), liver disease (OR = 1.84, 95% CI: 1.04–3.26, p = 0.04), liver cancer (OR = 3.91, 95% CI: 1.21–12.63, p = 0.02), joint replacement surgeries (OR = 1.93, 95% CI: 1.11–3.36, p = 0.02), and any outcome (OR = 1.59, 95% CI: 1.09–2.30, p = 0.01), although these were not statistically significant after correction for multiple testing (p ≤0.0014). PGS for hepcidin, sTfR, SF, and TIBC showed no statistically significant associations with outcomes (Table S10 and Fig. S2).

After excluding individuals with haemochromatosis at baseline, the only significant association after correction for multiple testing was that between serum iron PGS and haemochromatosis. Table S9 details the full list of associations in male C282Y homozygotes without a diagnosis of baseline haemochromatosis. Results remained consistent after excluding participants with relatedness to male C282Y homozygous participants (Table S11).

PGS and disease outcomes in female *HFE* C282Y homozygotes

Women with higher TSAT PGS had significantly increased odds of haemochromatosis diagnosis (OR per 1-SD increase = 1.33, 95% CI: 1.18–1.51, $p = 4.09 \times 10^{-6}$) compared with those without a diagnosis of haemochromatosis. Similarly, higher serum iron PGS was associated with increased odds of haemochromatosis (OR = 1.35, 95% CI: 1.20–1.53, $p = 1.60 \times 10^{-6}$). Hepcidin, sTfR, SF, and TIBC polygenic scores were not associated with outcomes after controlling for multiple tests ($p \leq 4 \times 10^{-6}$) (SI Table 12).

Women in the highest TSAT PGS quintile were more likely to be diagnosed with haemochromatosis (OR = 2.21, 95% CI: 1.50-3.25, p = 0.00006), compared to those with the lowest TSAT PGS quintile. The cumulative incidence by age 80 was 45.3% (95% CI: 36.9–52.7%) *vs.* 23.3% (17.3–28.8%) in the lowest quintile ($p = 1 \times 10^{-5}$) (Fig. 3A,B; Tables S13 and S14). In Fig. 3A, red points indicate associations that passed the BH FDR threshold ($p \leq 6 \times 10^{-5}$), orange points represent nominal significance (p <0.05), and grey points are not significant (p >0.05). The composite outcome analysed comprised liver disease, liver fibrosis/cirrhosis, and osteoarthritis. Cumulative incidence estimates for haemochromatosis were calculated from age 40 to 80 years. Equivalent plots excluding participants with prevalent haemochromatosis are shown in Fig. S4.

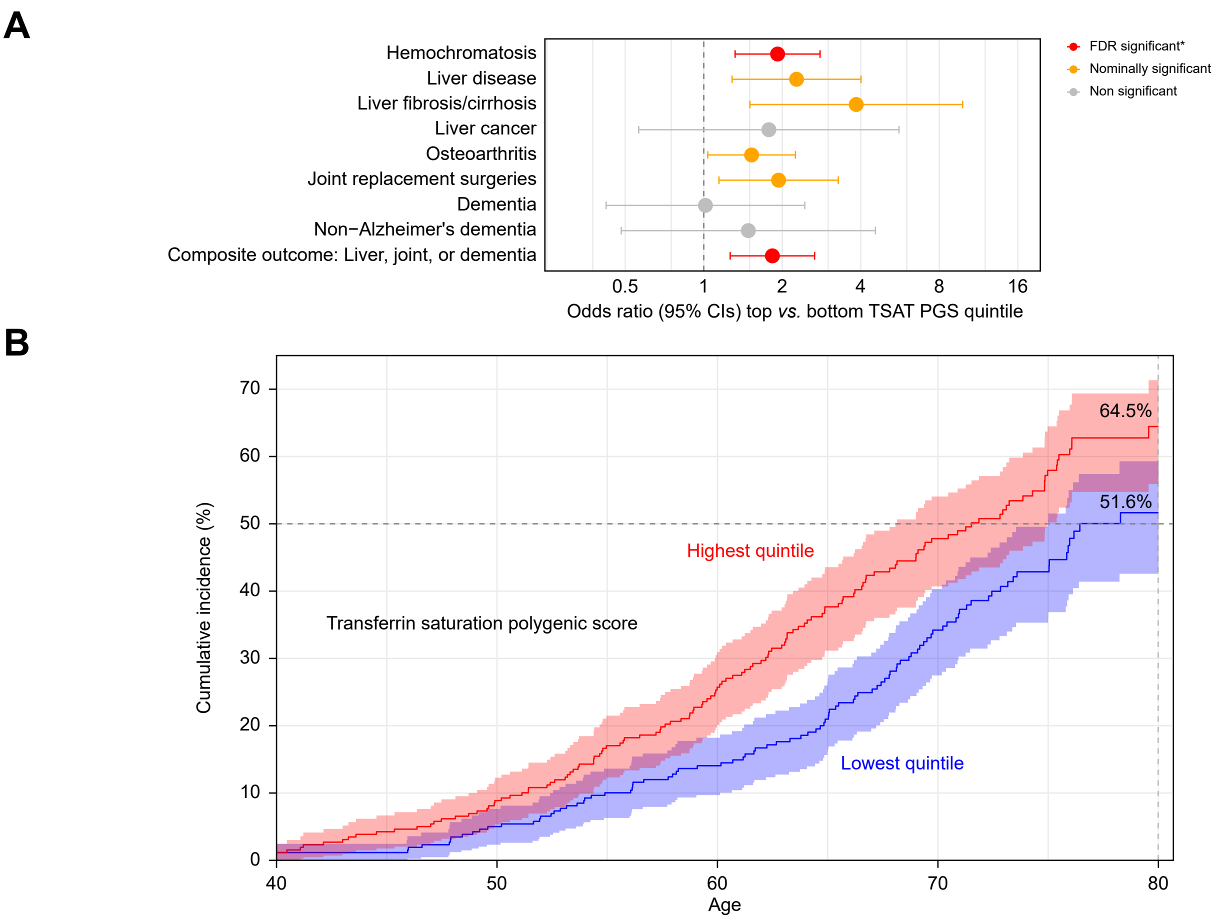

Fig. 2. Associations between high genetically predicted TSAT and clinical outcomes in male C282Y homozygotes. (A) Forest plot from logistic regression analyses in male C282Y homozygotes (n = 1,295) of associations between high genetically predicted TSAT (top PGS quintile) and risk of hospital-diagnosed clinical outcomes (*vs.* bottom PGS quintile). The composite outcome includes liver disease, liver fibrosis/cirrhosis, liver cancer, osteoarthritis, dementia, non-Alzheimer's dementia, and joint replacement surgeries. Red points indicate associations that passed the Benjamini–Hochberg FDR threshold (q <0.05), orange points represent nominal significance (*p* <0.05), and grey points are not significant. (B) Cumulative incidence for the composite outcome in the high and low-TSAT PGS groups, showing the cumulative incidence of diagnoses from age 40 to 80. See Fig. S1 for equivalent plots after excluding participants with diagnosed haemochromatosis. FDR, false discovery rate; PGS, polygenic score; TSAT, transferrin saturation.

Similarly, women in the highest serum iron PGS quintile had increased odds of being diagnosed with haemochromatosis (OR = 2.37, 95% CI: 1.57–3.57, p = 3.89 × 10^{-5}), with a cumulative incidence of 39.0% (95% CI: 31.5–45.7%) compared with 20.5% (13.9–26.5%) in the lowest quintile (p = 0.0001) (Tables S13 and S14; Fig. S3).

Hepcidin, sTfR, SF, and TIBC PGS showed no statistically significant associations with any outcomes (Table S13 and Fig. S3).

In female *HFE* C282Y homozygotes, the results were largely unchanged when excluding participants diagnosed with haemochromatosis before baseline assessment. Haemochromatosis remained the only outcome associated with the highest quintile of TSAT and serum iron PGS (see Table S15 for full results and Fig. S4 for TSAT associations without baseline haemochromatosis diagnosis). Results remained consistent after excluding female participants who were genetically related to other female C282Y homozygotes (Table S16).

Effect of serum iron and TSAT PGS on haemochromatosis incidence in non-C282Y homozygotes

In logistic regression analysis of outcome 'ever diagnosed with haemochromatosis', higher TSAT and serum iron PGS in H63D homozygous men significantly increased odds (TSAT OR per 1-SD = 2.11, 95% CI: 1.55–2.91, p = 6.00 × 10^{-5}; iron OR = 1.63, 95% CI: 1.21–2.23, p = 0.015). Associations in other *HFE* genotype groups (C282Y heterozygotes, etc.) were not statistically significant (FDR p >0.05). In analyses comparing highest *vs.* lowest quintiles of PGS, no associations were significant after multiple testing correction (FDR p >0.05).

We found similar estimates of cumulative incidence of haemochromatosis to age 80 between the top and bottom quintile of iron or TSAT PGS, in analyses stratified by *HFE* genotype. For example, among C282Y heterozygous men, cumulative incidence estimates were 0.6% (95% CI: 0.3–0.9) *vs.* 1.1% (95% CI: 0.7–1.5) for serum iron PGS (difference p = 0.178), and 0.6% (95% CI: 0.3–0.8) *vs.* 0.8% (95% CI: 0.4–1.1)

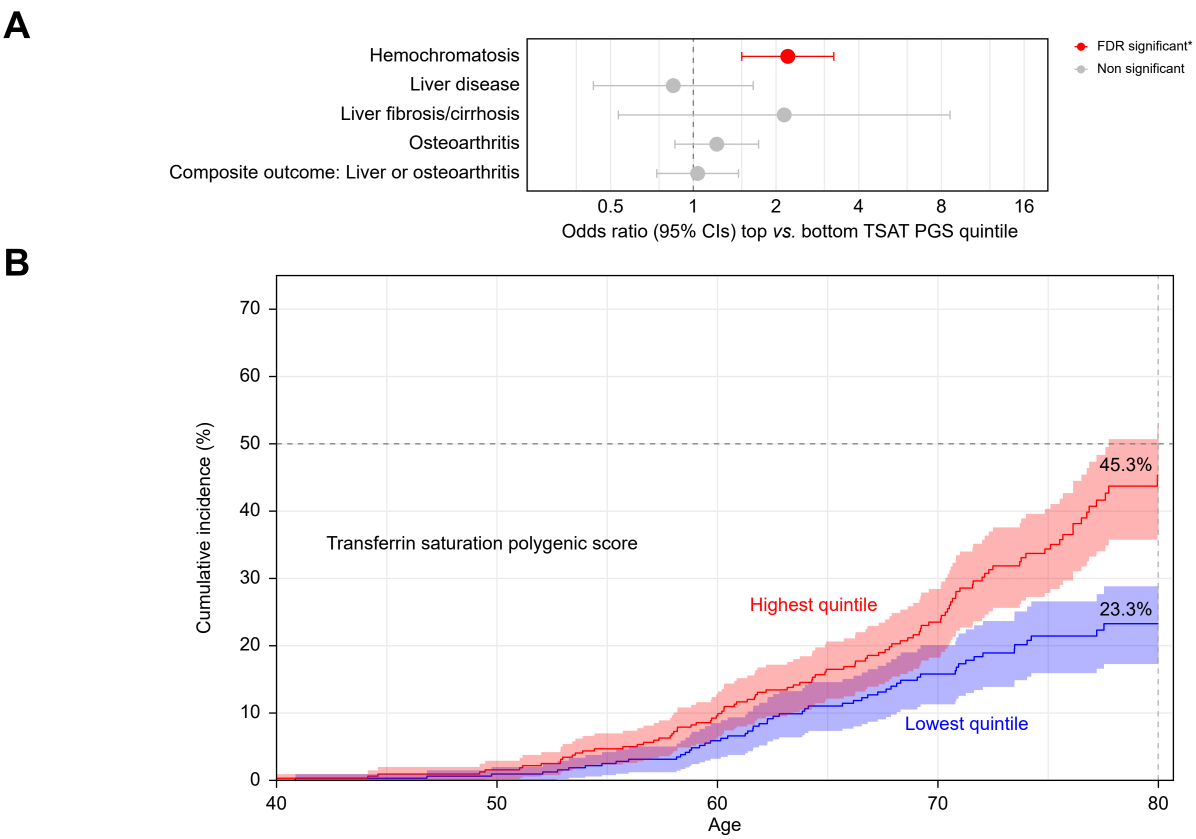

Fig. 3. Associations between high genetically predicted TSAT and clinical outcomes in female C282Y homozygotes. (A) Forest plot from logistic regression analyses in female C282Y homozygotes (n = 1,598) of associations between high genetically predicted TSAT (top PGS quintile) and risk of hospital-diagnosed clinical outcomes (*vs.* bottom PGS quintile). The composite outcome includes liver disease, liver fibrosis/cirrhosis, and osteoarthritis. Red points indicate associations that passed the Benjamini–Hochberg FDR threshold (q <0.05), orange points represent nominal significance (*p* <0.05), and grey points are not significant. (B) Cumulative incidence for haemochromatosis in the high and low -SAT PGS groups, showing the cumulative incidence of diagnoses from age 40 to 80. See Fig. S4 for equivalent plots after excluding participants with prevalent haemochromatosis. FDR, false discovery rate; PGS, polygenic score; TSAT, transferrin saturation.

for TSAT PGS (difference *p* = 0.279). These differences were not statistically significant. Similar trends of nonsignificant differences were seen across other *HFE* genotype groups (Table S17), and in analysis of female participants (difference *p* >0.05 for all comparisons).

Predictive models for disease outcomes

In time-to-event (Cox) models from the baseline assessment, we found that age, lifestyle factors, and genetics were statistically significant predictors for haemochromatosis and clinical outcomes among C282Y homozygotes (Table 2). Three nested models were compared: Model 1 (age only), Model 2 (age plus lifestyle factors, including alcohol intake, red or processed meat consumption, smoking status, WHR, and BMI), and Model 3 (age, lifestyle factors, and PGS). Among male C282Y homozygotes, model C-statistic increased from 0.51 (age only) to 0.58 with lifestyle factors (*p* = 0.003), and further to 0.60 with the addition of iron-related PGS, although the addition of PGS did not significantly improve discrimination (*p* = 0.14). In women, the impact of lifestyle was even more pronounced, with the C-statistic increasing from 0.51 (age only) to 0.64 (age + lifestyle; $p = 1.05 \times 10^{-7}$), and further to 0.68 when incorporating PGS (*p* = 0.02). For liver disease in women, the model C-statistic increased from 0.56 (age only) to 0.70 (age + lifestyle; *p* = 0.002), but remained at 0.70 with PGS (*p* = 0.74). A similar pattern was seen for any haemochromatosis-related clinical outcome, with female C-statistics improving from 0.61 (age) to 0.66 (lifestyle; $p = 7.28 \times 10^{-5}$) and to 0.66 with PGS (*p* = 0.43). See Table 2 and Table S18 for full C-statistics and model comparisons across outcomes.

Rare variants in *HFE* increased risk of haemochromatosis

In the analysis of 28,481 variants within 2 Mbp of *HFE* with minor allele count ≥10, we identified 8,829 statistically significant ($p < 5 \times 10^{-8}$) associations with haemochromatosis diagnosis (see Table S19 for single variant results and Fig. S5 for a Locus Zoom plot). Of these, 4,255 had low correlation (linkage disequilibrium [LD]) with C282Y genotype (R^2 <0.01): however, none remained significantly associated with haemochromatosis diagnosis after conditioning on C282Y genotype (nominal *p* >0.001), demonstrating the strength of association in the region resulting from cryptic LD with the known genotype.

In the aggregate variant analysis, we combined very rare variants (minor allele frequency <0.1%) with similar predicted functions together in 'masks', such as '*HFE* pLoF'. One mask was associated with haemochromatosis after conditioning on

Table 2. Predictive model comparison by outcome and sex in *HFE* C282Y homozygotes.

Sex	Model 1 (age)	Model 2 (age + lifestyle)	*p* value (M1 *vs.* M2)	Model 3 (M2 + PGS)	*p* value (M2 *vs.* M3)
Haemochromatosis					
Male	0.51	0.58	0.003	0.60	0.14
Female	0.51	0.64	1.05×10^{-7}	0.68	0.02
Liver disease					
Male	0.52	0.66	0.0003	0.69	0.31
Female	0.56	0.70	0.002	0.70	0.74
Any outcome*					
Male	0.61	0.64	0.04	0.64	0.48
Female	0.61	0.66	7.28×10^{-5}	0.66	0.43

Predictive performance (C-statistics from time-to-event [Cox] models) for incident diagnoses of haemochromatosis, any outcome, and liver disease among C282Y homozygotes. Three models were assessed: Model 1 includes age only; Model 2 adds lifestyle; and Model 3 further includes polygenic scores. Comparisons illustrate improvements in model discrimination, with *p* values provided for nested comparisons (Model 2 *vs.* Model 3) where available.

*The composite 'Any outcome' includes liver disease, liver fibrosis/cirrhosis, liver cancer, osteoarthritis, dementia, and joint replacement surgeries for male C282Y homozygotes, and liver disease, liver fibrosis/cirrhosis, and osteoarthritis for female C282Y homozygotes. These components were selected based on associations identified in our previous publication.[8] See supplementary data for full model results. PGS, polygenic score.

C282Y genotype: carriers of any of 31 rare *HFE* pLoF variants had increased likelihood of haemochromatosis diagnosis (OR = 14.8, 95% CI: 4.65–47.10, p = 0.003) (see Table S20 for aggregate results). Other masks, such as for rare missense variants, were not significantly associated with haemochromatosis (p >0.05). Among EUR-like genetic ancestry participants, 142 carried any of the pLoF variants in the haemochromatosis-associated mask. Three individuals with a pLoF variant in *HFE* had a diagnosis of haemochromatosis in the available data (two were heterozygous for *HFE* C282Y). One of these pLoF variants, three had previously been reported in ClinVar as pathogenic from a single source (chr6:26091040:AG:A is a single nucleotide deletion resulting in a frameshift: NM_000410.4[HFE]:c.279del [p.Trp94fs]). We found no associations between these rare variants and risk of liver disease, liver cancer, osteoarthritis, or dementia, although analyses for these outcomes were limited by very small case numbers among variant carriers (Table S21). Thirty of the 31 pLoF variants in the mask were in LD with *HFE* C282Y (D' = 1), although one was not (chr6:26090975:C:T D' = 0.07). For chr6:26091040:AG:A (the previously reported pathogenic pLoF variant), the estimated haplotype frequencies were consistent with the minor allele being out of phase with C282Y (*i.e.* not co-inherited on the same haplotype).

Discussion

Given the high prevalence of *HFE* C282Y in many populations and variation in clinical outcomes, it is important to identify those who might benefit from earlier or more targeted treatment. We demonstrated that non-*HFE* genetic variants influence haemochromatosis penetrance and expressivity. Male *HFE* C282Y homozygotes genetically predisposed to higher TSAT had a greater risk of haemochromatosis and severe clinical outcomes, such as liver and musculoskeletal complications, compared with those with genetically predicted low TSAT. This is similar to other conditions where PGS stratify patients by severity.[31,32] We also showed that including genetic, demographic, and lifestyle factors in predictive models gave the highest prediction accuracy, highlighting the multifactorial nature of haemochromatosis clinical penetrance. Clinically, integrating polygenic risk assessments with current screening protocols alongside routine *HFE* genetic testing could substantially enhance precision therapies for iron overload, enabling earlier identification of high-risk individuals and informing tailored interventions aimed at mitigating disease progression.

Rare pLoF variants in *HFE* increased the risk of haemochromatosis in non-C282Y homozygotes. This is consistent with *HFE* perturbations dysregulating hepcidin, thereby increasing iron absorption. This adds to the growing literature on rare genetic causes of iron overload.[33,34] Three pLoF variant carriers were diagnosed with haemochromatosis, where two were also heterozygous for C282Y: the estimated haplotype frequencies were consistent with trans-compound heterozygosity (the alleles being inherited separately). Although the ClinVar database contains numerous pLoF variants in *HFE* that are asserted as pathogenic for haemochromatosis, only one pLoF identified here was previously reported (chr6:26091040:AG:A). This highlights the need for further studies of rare causes of iron overload in non-C282Y homozygotes.

We extended previous work by incorporating: (1) longer health records follow-up (up to 30 October 2022 *vs*. 2021 previously); (2) PGS for hepcidin and sTfR; (3) additional clinical outcomes (associated with C282Y homozygosity in our recent study[8]); (4) cumulative incidence estimates for clinical diagnoses up to age 80 stratified by iron PGS; (5) combined clinical prediction modelling; and (6) analysis of rare variants in *HFE*. Higher genetically predicted TSAT was the most consistent predictor of adverse clinical outcomes in C282Y homozygotes. Elevated TSAT is the most sensitive and early indicator of haemochromatosis, although it is subject to biological variability and lacks specificity.[35] Previous reports suggested that persistently elevated TSAT are associated with worse outcomes.[36] Nevertheless, there is broad acknowledgment that venesection/phlebotomy treatment is less effective at addressing this aspect of the disease than it is in reducing stored iron (as reflected by SF), highlighting the pressing clinical need to understand the causal mechanisms in haemochromatosis.

In our previous analysis of iron PGS, we observed modest associations with haemochromatosis across several non-C282Y *HFE* genotypes in men, including H63D homozygotes and C282Y heterozygotes.[11] In the current study, we found no significant excess haemochromatosis cumulative incidence up to age 80 in participants with high TSAT or serum iron PGS within any *HFE* genotype group except C282Y homozygotes. These findings suggest that, at the population level, common non-*HFE* serum iron-associated genetic variants only meaningfully impact haemochromatosis incidence in *HFE* C282Y

homozygotes. This is especially evident in men, where haemochromatosis cumulative incidence reached 55.4% in the highest TSAT PGS quintile compared with 38.0% in the lowest.

While both serum iron and TSAT PGS were significantly associated with a haemochromatosis diagnosis in both male and female *HFE* C282Y homozygotes in our 2022 paper, their associations with broader morbidity, including joint replacement surgeries, dementia, and non-Alzheimer's dementia, were not previously analysed. In the current study, both TSAT and serum iron PGS were associated with increased likelihood of liver complications, with stronger and more consistent effects observed for TSAT within male C282Y homozygotes. We found that TSAT and serum iron PGSs are highly correlated because of partially overlapping genetic variants. Clinically, TSAT is preferred over serum iron because it better reflects available circulating iron and is less affected by short-term variation,[35,37] supporting its prioritisation as the primary polygenic modifier in risk models. The stronger effect estimates of the TSAT PGS highlight its value in predicting liver and musculoskeletal outcomes in male C282Y homozygotes.

Another notable finding was the absence of strong associations with dementia outcomes. Although a previous UK Biobank study reported increased risk of dementia associated with excess iron,[22] also within male C282Y homozygotes,[7] neither TSAT nor serum iron PGSs were predictive of these outcomes. This could reflect differing biological mechanisms or limited case numbers, suggesting that iron-related polygenic effects primarily influence earlier-stage rather than end-stage complications.[7,8]

Despite the robust associations between TSAT and serum iron PGSs and clinical outcomes, several PGS, including those for SF, TIBC, sTfR, and hepcidin, showed minimal or no associations. These findings are consistent with those from our previous study.[11] The weaker performance of these PGS biomarkers could reflect their susceptibility to nongenetic influences. SF is an acute-phase reactant,[38] and TIBC levels can inversely reflect iron burden, but lack direct causality in iron toxicity.[39]

The hepcidin PGS was also not robustly associated with clinical outcomes. While hepcidin is a key regulator of systemic iron homeostasis[40] and a target of emerging therapeutics (*e.g.* rusfertide[41]), the PGS used in this analysis was derived from a GWAS of serum hepcidin levels, which might not fully capture the regulatory activity of hepcidin within specific tissues. The hepcidin PGS was also not robustly associated with outcomes. Although hepcidin is a key regulator of systemic iron homeostasis,[40] the PGS used here might not fully capture regulatory activity within specific tissues, and limited GWAS sample sizes might have reduced power.[26] In the context of *HFE* haemochromatosis, where *HFE* and TMPRSS6 are major drivers of downstream hepcidin suppression, genetic variation in hepcidin itself could have a smaller role in modulating disease risk.

Predicting disease outcomes in haemochromatosis remains an important challenge. Combining genotypic risk factors with lifestyle factors, and possibly the development of more sensitive biomarkers of disease, offers the most comprehensive approach to identifying individuals at greatest lifetime risk and who would benefit most from targeted treatment.

Strengths and limitations

A major strength of this study is the large sample of *HFE* C282Y homozygotes, enabling robust sex-stratified estimates and cumulative incidence modeling of clinical outcomes. Extended follow-up of hospital inpatient diagnoses allowed assessment of penetrance to clinical outcomes up to age 80 years. Compared with our previous publication, this study includes more outcome events and additional PGS, offering broader insight into genetic modifiers of clinical risk in haemochromatosis and related outcomes.

This is also the first study to evaluate PGS for hepcidin and sTfR in relation to clinical outcomes specifically among C282Y homozygotes. Although associations for these markers were generally weak, their inclusion provides a broader view of the potential regulatory pathways influencing penetrance. However, limitations remain. The hepcidin score was based on circulating serum levels rather than tissue-specific activity, which could limit interpretation. Whereas predictive models improved when polygenic and lifestyle factors were included, overall discrimination remained moderate, and findings will require replication in other community-based cohorts. Lastly, the GWAS studies that identified the variants used to derive the iron-status PGS were conducted in general EUR ancestry populations and did not specifically include *HFE* C282Y homozygotes.[26,27] Thus, we assume that the genetic effects observed in these broader populations are consistent within C282Y homozygotes.

The generalisability of findings might be limited by the UK Biobank healthier volunteer bias and incomplete primary care data coverage, which could lead to underestimation of clinical penetrance. Although associations with TSAT and serum iron PGS were consistent and biologically plausible, predictive performance remained moderate and, thus, external validation will be needed. In addition, we did not have direct measures of TSAT in UK Biobank data and, therefore, we could not evaluate whether the TSAT PGS provides additional clinical value beyond actual blood measurements. Lastly, the hepcidin PGS, based on serum levels, might not fully capture its regulatory role in iron metabolism.

PGS for TSAT and lifestyle factors, such as alcohol intake, diet, smoking, and obesity, independently influence clinical penetrance in *HFE* C282Y homozygous individuals. Our findings highlight the potential for improved screening strategies to identify high-risk individuals and also for monitoring strategies for targeted management of iron overload in *HFE* C282Y homozygotes. Finally, rare, large-effect variants in *HFE* might reflect novel causes of iron overload in non-C282Y homozygotes.

Affiliations
[1]*Department of Clinical and Biomedical Sciences, University of Exeter, Exeter, UK;* [2]*Department of Gastroenterology, South Warwickshire NHS Foundation Trust, Warwick, UK;* [3]*Warwick Medical School, Warwick, UK*

Abbreviations

BH, Benjamini–Hochberg; EUR: European; FDR, false discovery rate; GWAS, genome-wide association study; HCC, hepatocellular carcinoma; LD, linkage disequilibrium; OR, odds ratio; PC, principial component; PGS, polygenic score; pLOF, predicted loss-of-function; SF, serum ferritin; sTfR, serum transferrin receptor; TIBC, total iron-binding capacity; TSAT, transferrin saturation; VIF, variance inflation factor; WHR, waist–hip ratio.

Financial support

JA has an NIHR Advanced Fellowship (NIHR301844). The University of Exeter supports ML and LP. GH is supported by the Medical Research Council (no. UKRI327). This work was also supported by a Medical Research Council project grant (no. UKRI2536).

Conflicts of interest

JDS received consulting fees from CSL Behring for advice on haemochromatosis treatments. All other authors have no conflicts of interest to declare.
Please refer to the accompanying ICMJE disclosure forms for further details.

Authors' contributions

ML: conceptualization, data curation, formal analysis, investigation, visualization, writing – original draft. JD: methodology, writing – original draft. RB: data curation, formal analysis, investigation, writing – review and editing. GH, AW: data curation, software, writing – review and editing. CW: supervision, writing – review and editing. JS: conceptualization, writing – original draft preparation. JA: conceptualization, funding acquisition, investigation, methodology, supervision, writing – original draft preparation, writing - review and editing. LP: conceptualization, funding acquisition, data curation, formal analysis, investigation, methodology, project administration, supervision, writing - original draft preparation, writing - review and editing.

Data availability

Data are available on application to the UK Biobank (www.ukbiobank.ac.uk/register-apply). We have published an R package for working in the UK Biobank Research Analysis Platform (https://github.com/lcpilling/ukbrapR), which can be used with the clinical codes and genetic variant information in our supplementary data to create the genetic and health outcome phenotypes.

Acknowledgements

This research was conducted using the UK Biobank resource, under applications 14631 and 103356. We thank the UK Biobank participants and coordinators for the dataset. This work used data provided by patients and collected by the NHS as part of their care and support. Copyright © (2025), NHS England. Re-used with the permission of the NHS England and UK Biobank. All rights reserved. This research also used data assets made available by National Safe Haven as part of the Data and Connectivity National Core Study, led by Health Data Research UK in partnership with the Office for National Statistics and funded by UK Research and Innovation. This study was supported by the National Institute for Health and Care Research Exeter Biomedical Research Center. The views expressed are those of the authors and not necessarily those of the NIHR or the Department of Health and Social Care. For the purpose of open access, the authors have applied a 'Creative Commons Attribution (CC BY)' license to any Author Accepted Manuscript version arising from this submission.

Supplementary data

Supplementary data to this article can be found online at https://doi.org/10.1016/j.jhepr.2026.101781.

References

Author names in bold designate shared co-first authorship

[1] Katsarou M-S, Papasavva M, Latsi R, et al. Hemochromatosis: hereditary hemochromatosis and HFE gene. Vitam Horm 2019;110:201–222.
[2] Broad Institute. gnomAD browser: variant 6-26092913-G-A (GRCh38). GnomAD v410 2024. https://gnomad.broadinstitute.org/variant/6-26092913-G-A?dataset=gnomad_r4. [Accessed 16 February 2026].
[3] Fairbanks V, Barton JC, Edwards CQ. Hemochromatosis: population genetics. In: Barton GC, Edwards CQ, editors. Hemochromatosis: genetics, pathophysiology, diagnosis and treatment. Cambridge: Cambridge University Press; 2000. p. 42–50.
[4] Adams PC, Jeffrey G, Ryan J. Haemochromatosis. Lancet 2023;401:1811–1821.
[5] Atkins JL, Pilling LC, Masoli JAH, et al. Association of hemochromatosis *HFE* p.C282Y homozygosity with hepatic malignancy. JAMA 2020;324:2048.
[6] Banfield LR, Knapp KM, Pilling LC, et al. Hemochromatosis genetic variants and musculoskeletal outcomes: 11.5-year follow-Up in the UK Biobank Cohort Study. JBMR Plus 2023;7.
[7] Atkins JL, Pilling LC, Heales CJ, et al. Hemochromatosis mutations, brain iron imaging, and dementia in the UK Biobank Cohort. J Alzheimers Dis 2021;79:1203–1211.
[8] **Lucas MR**, **Atkins JL**, Pilling LC, et al. *HFE* genotypes, haemochromatosis diagnosis and clinical outcomes at age 80 years: a prospective cohort study in the UK Biobank. BMJ Open 2024;14:e081926.
[9] Beutler E, Felitti VJ, Koziol JA, et al. Penetrance of 845G→ A (C282Y) HFE hereditary haemochromatosis mutation in the USA. Lancet 2002;359:211–218.
[10] Allen KJ, Gurrin LC, Constantine CC, et al. Iron-overload-related disease in *HFE* hereditary hemochromatosis. N Engl J Med 2008;358:221–230.
[11] Pilling LC, Atkins JL, Melzer D. Genetic modifiers of penetrance to liver endpoints in HFE hemochromatosis: associations in a large community cohort. Hepatology 2022;76:1735–1745.
[12] Lucas MR, Pilling LC, Delgado J, et al. Liver iron levels are associated with *HFE* -hemochromatosis genotype, diet, adiposity, and disease in the UK Biobank 2025. Hepatol Commun 2026;10:e0883.
[13] Radio FC, Majore S, Aurizi C, et al. Hereditary hemochromatosis type 1 phenotype modifiers in Italian patients. the controversial role of variants in HAMP, BMP2, FTL and SLC40A1 genes. Blood Cells Mol Dis 2015;55:71–75.
[14] Rochette J, Le Gac G, Lassoued K, et al. Factors influencing disease phenotype and penetrance in HFE haemochromatosis. Hum Genet 2010;128:233–248.
[15] Milet J, Déhais V, Bourgain C, et al. Common variants in the BMP2, BMP4, and HJV genes of the hepcidin regulation pathway modulate HFE hemochromatosis penetrance. Am J Hum Genet 2007;81:799–807.
[16] Altès A, Bach V, Ruiz A, et al. Does the SLC40A1 gene modify HFE-related haemochromatosis phenotypes? Ann Hematol 2009;88:341–345.
[17] Galesloot TE, Geurts-Moespot AJ, den Heijer M, et al. Associations of common variants in *HFE* and *TMPRSS6* with iron parameters are independent of serum hepcidin in a general population: a replication study. J Med Genet 2013;50:593–598.
[18] Benyamin B, McRae AF, Zhu G, et al. Variants in TF and HFE explain approximately 40% of genetic variation in serum-transferrin levels. Am J Hum Genet 2009;84:60–65.
[19] Babitt JL, Huang FW, Wrighting DM, et al. Bone morphogenetic protein signaling by hemojuvelin regulates hepcidin expression. Nat Genet 2006;38:531–539.
[20] Fry A, Littlejohns TJ, Sudlow C, et al. Comparison of sociodemographic and health-related characteristics of UK Biobank participants with those of the general population. Am J Epidemiol 2017;186:1026–1034.
[21] National Academies of Sciences, Engineering and Medicine. Using population descriptors in genetics and Genomics research: a new framework for an evolving field. Washington, DC: National Academies Press; 2023.
[22] **Casanova F**, **Tian Q**, Atkins JL, et al. Iron and risk of dementia: mendelian randomisation analysis in UK Biobank. J Med Genet 2024;61:435–442.
[23] Van Hout CV, Tachmazidou I, Backman JD, et al. Exome sequencing and characterization of 49,960 individuals in the UK Biobank. Nature 2020;586:749–756.
[24] WHO. Waist circumference and waist-hip ratio: report of a WHO expert consultation. Geneva: World Health Organization; 2008.
[25] Bradbury KE, Murphy N, Key TJ. Diet and colorectal cancer in UK Biobank: a prospective study. Int J Epidemiol 2020;49:246–258.
[26] **Allara E**, **Bell S**, Smith R, et al. Novel loci and biomedical consequences of iron homoeostasis variation. Commun Biol 2024;7:1631.
[27] Moksnes MR, Graham SE, Wu K-H, et al. Genome-wide meta-analysis of iron status biomarkers and the effect of iron on all-cause mortality in HUNT. Commun Biol 2022;5:591.
[28] Maher BS. Polygenic scores in epidemiology: risk prediction, etiology, and clinical utility. Curr Epidemiol Rep 2015;2:239–244.
[29] Hellwege JN, Keaton JM, Giri A, et al. Population stratification in genetic association studies. Curr Protoc Hum Genet 2017;95(1.22):1–1.22.23.

[30] Shimonovich M, Pearce A, Thomson H, et al. Assessing causality in epidemiology: revisiting Bradford Hill to incorporate developments in causal thinking. Eur J Epidemiol 2021;36:873–887.
[31] **Fahed AC**, **Wang M**, **Homburger JR**, et al. Polygenic background modifies penetrance of monogenic variants for tier 1 genomic conditions. Nat Commun 2020;11:3635.
[32] Gallagher S, Hughes E, Wagner S, et al. Association of a polygenic risk score with breast cancer among women carriers of high- and moderate-risk breast cancer genes. JAMA Netw Open 2020;3:e208501.
[33] Ouyang Q, Li Y, Xu A, et al. Recurrent BMP4 variants in exon 4 cause non-HFE-associated hemochromatosis via the BMP/SMAD signaling pathway. Orphanet J Rare Dis 2024;19:429.
[34] **Zhang W**, **Li Y**, Xu A, et al. Identification of novel non-HFE mutations in Chinese patients with hereditary hemochromatosis. Orphanet J Rare Dis 2022;17:216.
[35] European Association for the Study of the Liver. EASL clinical practice guidelines on haemochromatosis. J Hepatol 2022;77:479–502.
[36] Bardou-Jacquet E, Morcet J, Manet G, et al. Decreased cardiovascular and extrahepatic cancer-related mortality in treated patients with mild HFE hemochromatosis. J Hepatol 2015;62:682–689.
[37] Pfeiffer CM, Looker AC. Laboratory methodologies for indicators of iron status: strengths, limitations, and analytical challenges. Am J Clin Nutr 2017;106:1606S–1614S.
[38] Jehn M, Clark JM, Guallar E. Serum ferritin and risk of the metabolic syndrome in U.S. adults. Diabetes Care 2004;27:2422–2428.
[39] Siff JE, Meldon SW, Tomassoni AJ. Usefulness of the total iron binding capacity in the evaluation and treatment of acute iron overdose. Ann Emerg Med 1999;33:73–76.
[40] Kwapisz J, Slomka A, Zekanowska E. Hepcidin and its role in iron homeostasis. EJIFCC 2009;20:124–128.
[41] Kowdley KV, Modi NB, Peltekian K, et al. Rusfertide for the treatment of iron overload in HFE-related haemochromatosis: an open-label, multicentre, proof-of-concept phase 2 trial. Lancet Gastroenterol Hepatol 2023;8:1118–1128.

Keywords: Hereditary haemochromatosis; *HFE* gene; Iron overload; Polygenic risk scores; Liver disease; Genetic modifiers.
Received 24 September 2025; received in revised form 27 January 2026; accepted 3 February 2026; Available online 13 February 2026

Journal of Hepatology, Volume *8*

Supplemental information

Genetic and lifestyle modifiers of haemochromatosis-related clinical outcomes in *HFE* C282Y homozygotes

Mitchell R. Lucas, João Delgado, Robin N. Beaumont, Gareth Hawkes, Andrew R. Wood, Caroline F. Wright, Jeremy D. Shearman, Janice L. Atkins, and Luke C. Pilling

Genetic and lifestyle modifiers of haemochromatosis-related clinical outcomes in *HFE* C282Y homozygotes

Mitchell R Lucas, João Delgado, Robin N Beaumont, Gareth Hawkes, Andrew R Wood, Caroline F. Wright, Jeremy D Shearman, Janice L Atkins, Luke C Pilling

Table of contents

Supplementary methods 2

Supplementary tables... separate excel file

Fig. S1 4

Fig. S2 5

Fig. S3 6

Fig. S4 7

Fig. S5 8

Supplementary methods

Polygenic Score for Iron Biomarkers

Supplementary Table 2 includes the genetic variants used to create the polygenic scores for each trait. The position is in human genome build 38. We used R package {ukbrapR} v0.3.5 to create the polygenic scores in the UK Biobank Research Analysis Platform (https://github.com/lcpilling/ukbrapR). This uses `bgenix` (https://enkre.net/cgi-bin/code/bgen/doc/trunk/doc/wiki/bgenix.md) to extract the variant calls from the UK Biobank whole genome sequencing DRAGEN variant calls (BGEN format, field:24309). Then `plink2` (https://www.cog-genomics.org/plink/2.0/) converts the subsetted BGEN file to BED format. Then `plink` v1.9 allele scoring function (https://www.cog-genomics.org/plink/1.9/score) is used to create the weighted allele score.

Baseline assessment (2006-2010)

We calculated body mass index (BMI) as weight (kg, field: 21002) divided by height (m^2, field: 50), waist-hip-ratio (WHR) by dividing waist circumference (cm, field: 48) by hip circumference (cm, field: 49). WHR was categorized into a binary variable based on sex-specific thresholds for central obesity.(24) Participants with WHR ≥0.96 (males) or WHR ≥0.85 (females) were assigned a value of 1, indicating high WHR, while those below these thresholds were assigned 0. Alcohol intake (fields: 1568 [weekly red wine], 1578 [weekly white wine/champagne], 1588 [weekly beer/cider], 1598 [weekly spirits], 1608 [weekly fortified wine], 4407 [monthly red wine], 4418 [monthly white wine/champagne], 4429 [monthly beer/cider], 4440 [monthly spirits], 4451 [monthly fortified wine], 4462 [monthly other alcoholic drinks], 5364 [weekly other alcoholic drinks]) was categorised by the number of units drank per week groups into '0 units per week', 1-14 units per week' (reference group, based on UK national recommendations1), '15-29 units per week' and 'over 30 units per week'. Smoking status (field: 20116) was defined as 'current smoker' based on subjects being asked "Do you smoke tobacco now? And grouped in a binary variable of 0 = no and 1 = yes, on most or all days' and 'Only occasionally'. Each participant was asked separate questions about their meat consumption during the assessment. They were specifically asked how often they ate processed meat (field: 1349), lamb/mutton (field: 1379), pork (field: 1389), and beef (field: 1369). For each type of meat, participants could choose from the following options: 'Never,' 'Less than once a week,' 'Once a week,' '2-4 times a week,' '5-6 times a week,' or 'Once or more daily'. We provided a value for each respective response on meat consumption: (Never = 0) (Less than once a week = 0.5) (Once a week = 1) (2-4 times a week = 3) (>4 times a week = 5.5) then derived a summed total weekly consumption of red /processed meat and recorded

as follow ‘0 times/week’ (reference group) and ‘0.1-2.9 times/week’, and ‘≥3.0 times/week’.(25) Missing assessment data were not imputed: complete-case analyses were used throughout.

Fig. S1

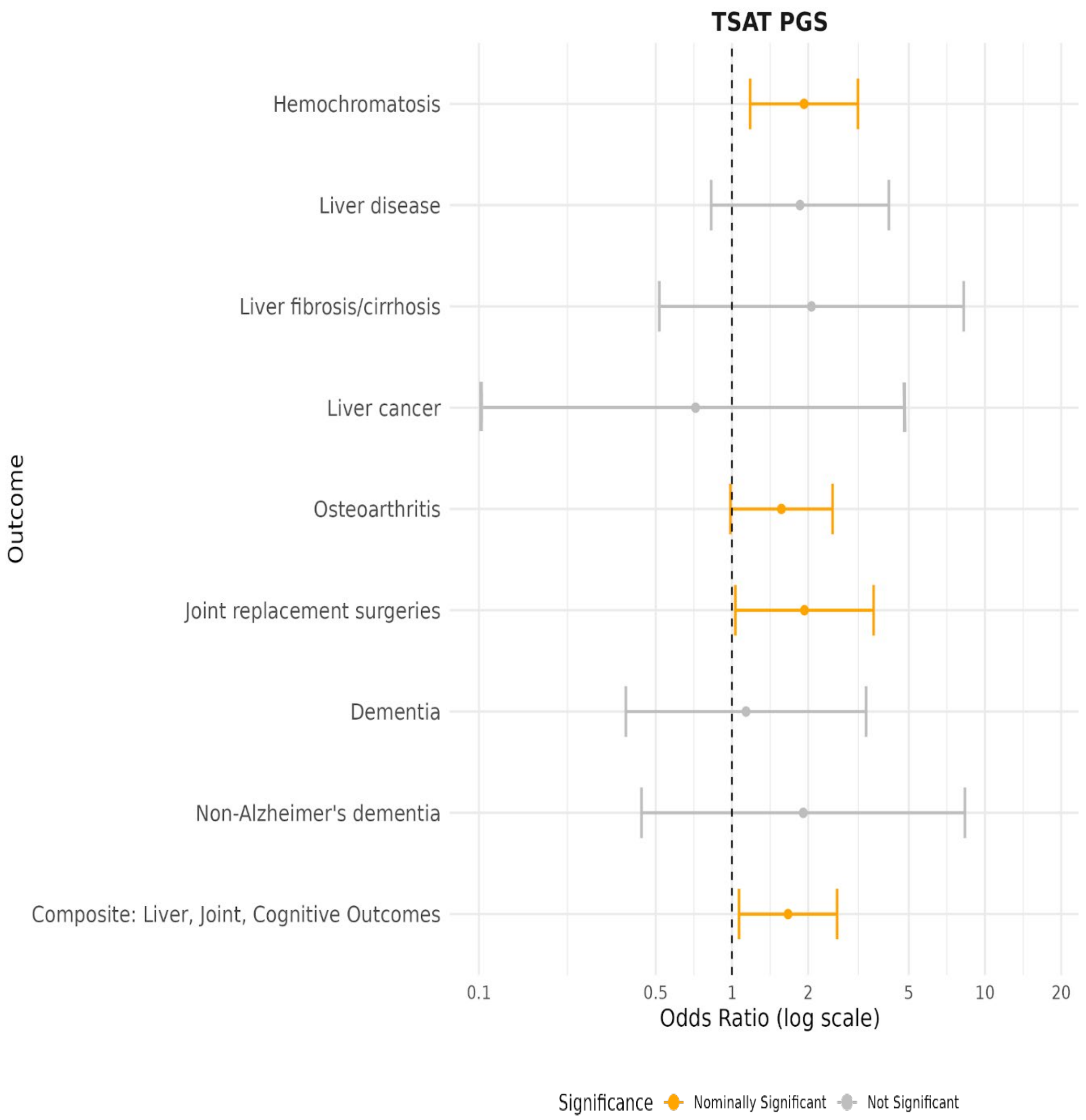

Associations between the highest quintile of TSAT polygenic biomarker and outcomes in C282Y homozygous male UK Biobank participants, without baseline hemochromatosis

Fig. S2

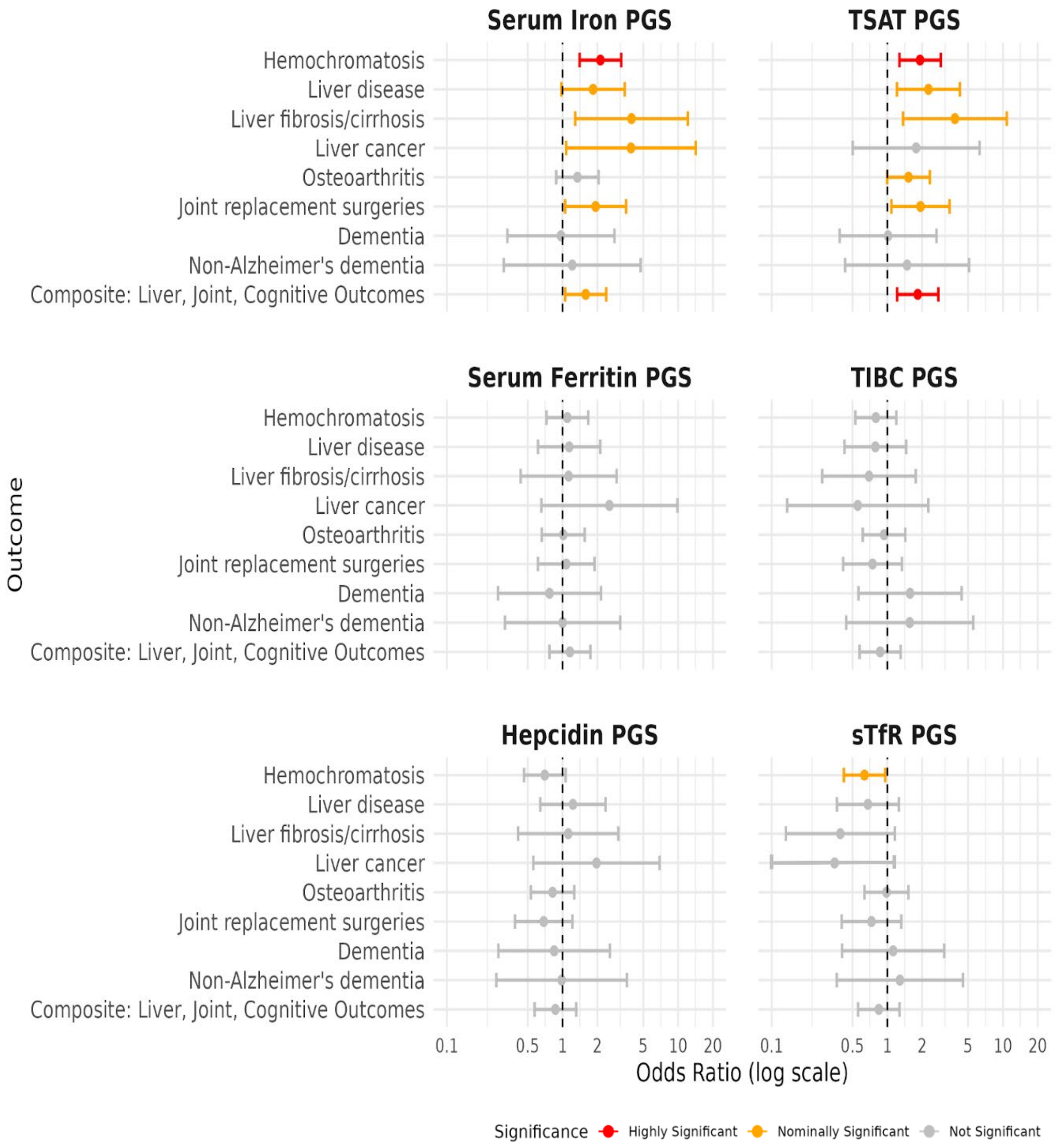

Associations between the highest quintile of iron polygenic biomarkers and outcomes in C282Y homozygous male UK Biobank participants.

Fig. S3

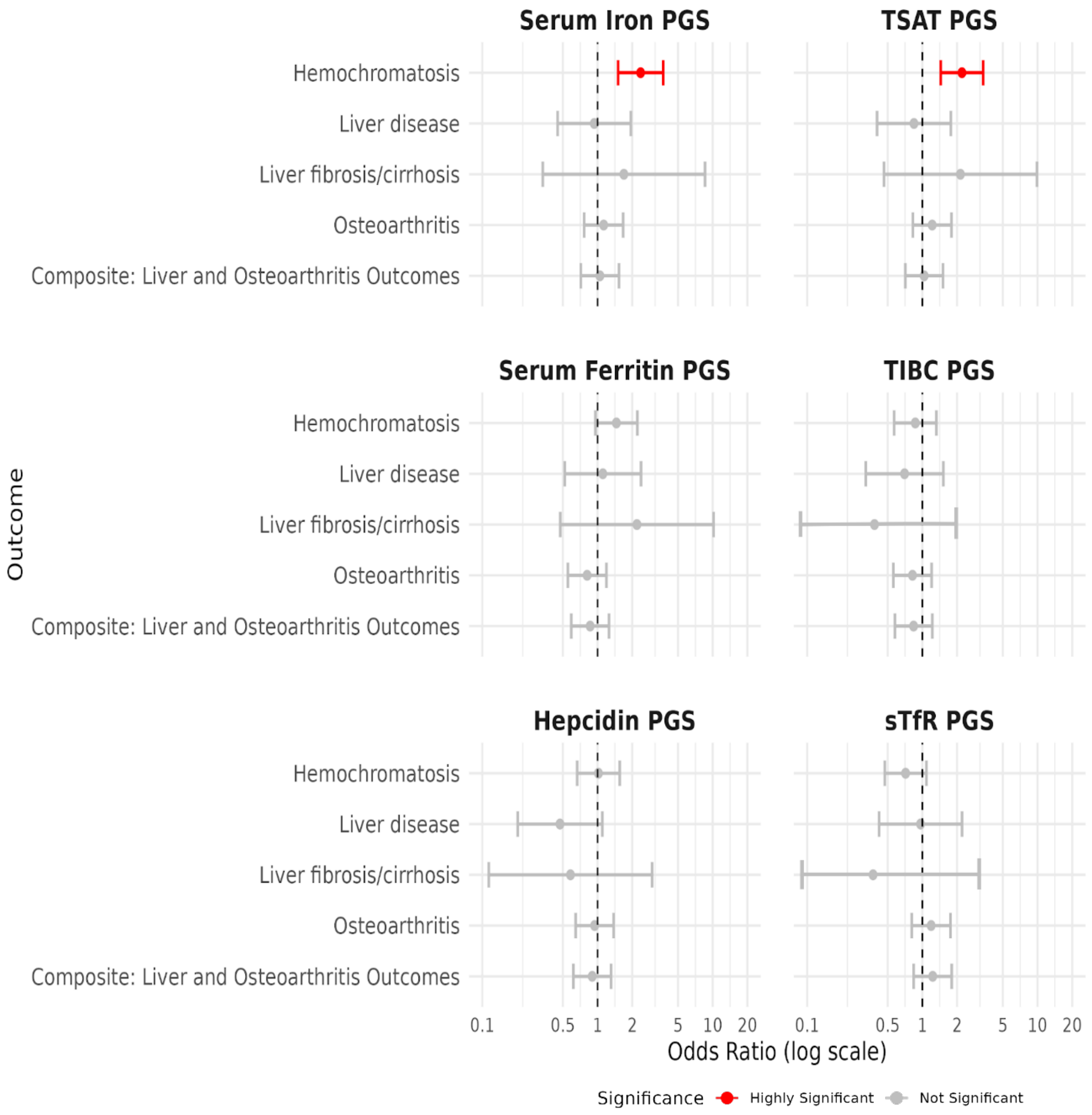

Associations between the highest quintile of iron polygenic biomarkers and outcomes in C282Y homozygous female UK Biobank participants

Fig. S4

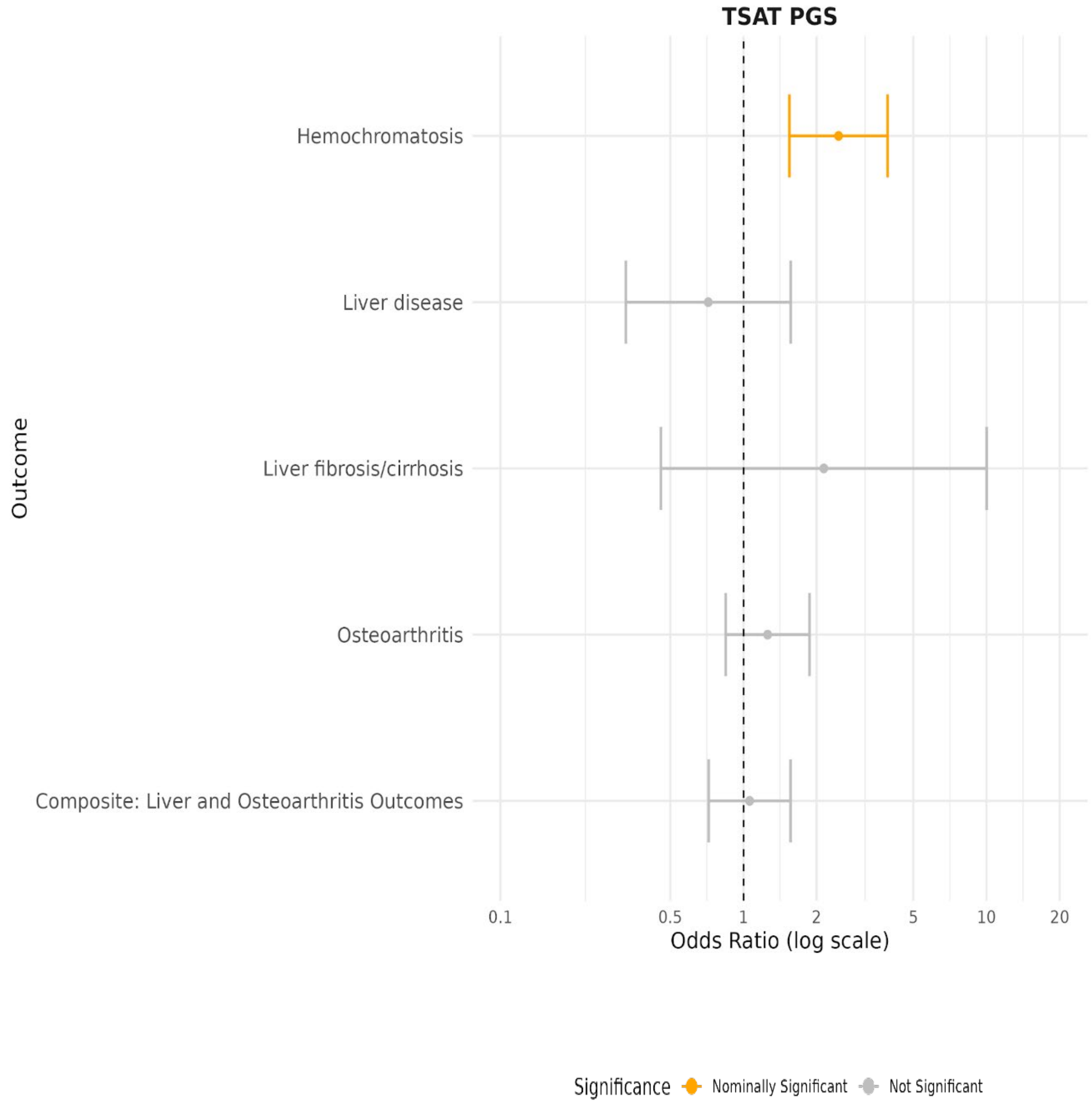

Associations between the highest quintile of TSAT polygenic biomarker and outcomes in C282Y homozygous female UK Biobank participants, without baseline hemochromatosis.

Fig. S5

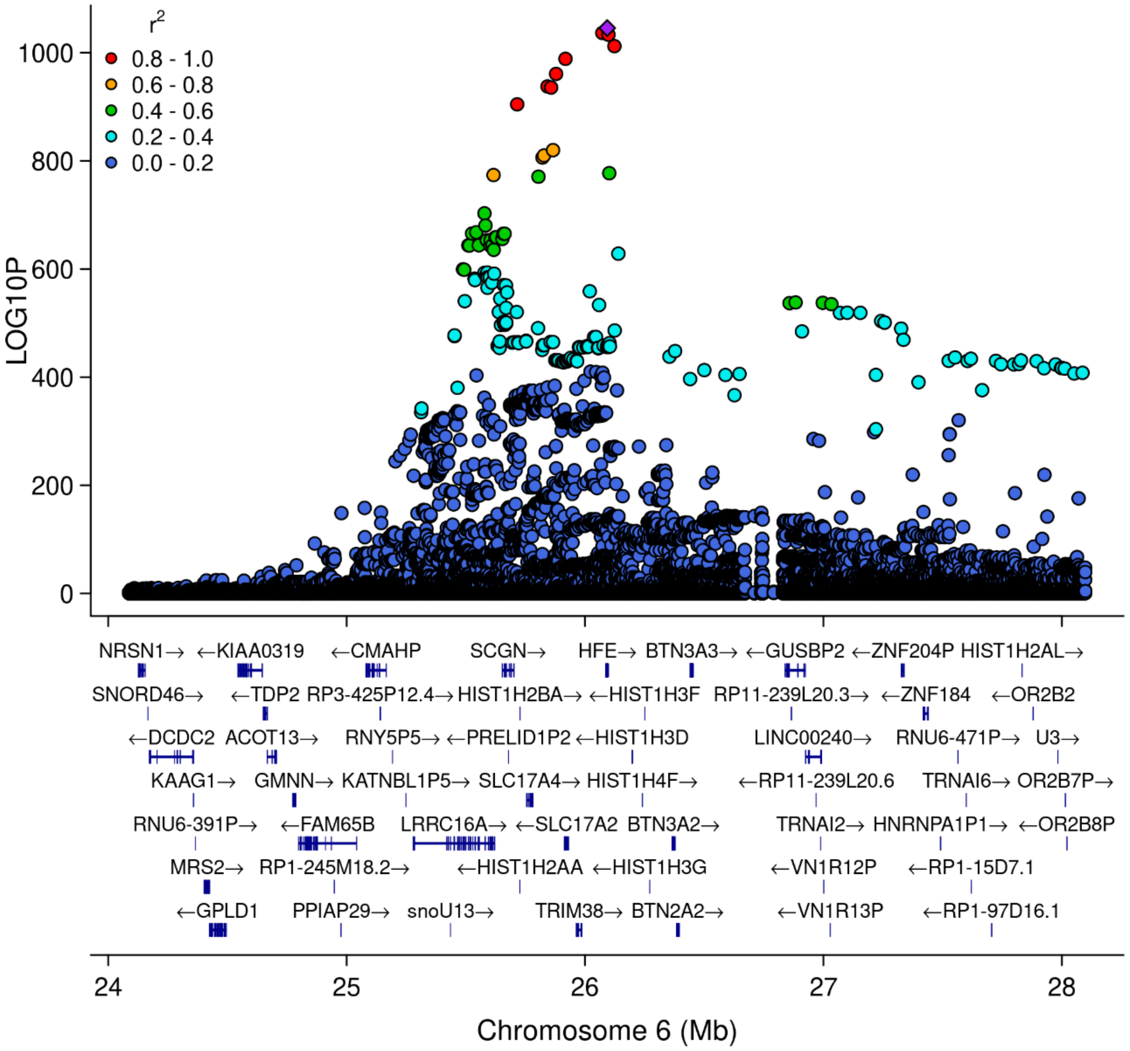

LocusZoom plot of *HFE* region on chromosome 6. Each point is a genetic variant: the x-axis shows it's location on chromosome 6, and the y-axis is the strength of association with haemochromatosis diagnosis (-$\log_{10}$ *p*-value). The points are coloured by their correlation coefficient (R^2) with *HFE* C282Y (rs1800562), coloured purple, in the UK Biobank EUR-like participants. Gene names and positions are indicated below the points, for reference. R package {locuszoomr} v0.3.8 was used to generate the plot (https://github.com/myles-lewis/locuszoomr).
